# Spatial differentiation and potential influencing factors of land-surface heat exposure based on street-view imagery: a case study of Luolong District, Luoyang

**DOI:** 10.3389/fpubh.2026.1960954

**Published:** 2026-10-07

**Authors:** Junhui Hu, Xinyao Zhang, Danfeng Liu, Luyao Chen

**Affiliations:** School of Civil Engineering and Architecture, Henan University of Science and Technology, Luoyang, Henan, China

**Keywords:** land-surface heat exposure, MGWR, street view imagery, street-view metrics, XGBoost-SHAP

## Abstract

**Introduction:**

Against the backdrop of increasingly frequent extreme heat events, mitigating land-surface heat exposure on urban streets is crucial for safeguarding public health. Existing studies have often overlooked the spatially differentiated effects of complex street environments on land-surface heat exposure and the associated cooling thresholds. Therefore, this study takes Luolong District, Luoyang, as a case study to investigate the spatially heterogeneous effects and nonlinear threshold characteristics of street-view metrics on land-surface heat exposure.

**Methods:**

This study integrates Baidu Street View imagery with multi-source spatial data and employs Multiscale Geographically Weighted Regression (MGWR) and the XGBoost-SHAP model to systematically investigate the spatially heterogeneous effects and nonlinear threshold characteristics of micro-scale street-view metrics on land-surface heat exposure, and proposes differentiated optimization strategies accordingly.

**Results:**

First, the effects of street morphology on land-surface heat exposure exhibit significant spatial multi-scale differentiation. Road proportion (RDP) and sidewalk proportion (SWP) operate predominantly at the micro scale, whereas the Green View Index (GVI) and Building Coverage Ratio (BCR) exhibit predominantly meso-scale effects; in contrast, the Sky View Factor (SVF) and Enclosure (ENC) demonstrate predominantly macro-scale effects. Second, nonlinear “threshold effects” and saturation points are generally observed between individual street-view metrics and land-surface heat exposure. For example, the cooling benefits of GVI are subject to an upper limit, whereas excessively high SVF values exhibit a strong positive association with increased land-surface heat exposure. Third, the risk classification and targeted renewal strategies developed based on a “current condition–sensitivity matrix” can effectively mitigate localized land-surface heat exposure across streets with different functional characteristics.

**Discussion:**

This study provides scientific decision-making support for the fine-grained renewal of high-density urban living environments and climate-adaptive urban governance.

## 1 Introduction

Under the dual pressures of global climate change and rapid urbanization, the increasing frequency of extreme heat events and the intensification of the urban heat island effect have emerged as major public health challenges threatening the health of populations living in high-density urban environments (1). Prolonged heat exposure not only increases the incidence of cardiovascular and respiratory diseases but also substantially elevates the risk of heatstroke and related mortality (2). These risks are particularly severe for climate-vulnerable populations, including older adults, children, and outdoor workers, who face considerable threats to their health and lives (3). Accordingly, many countries and multilateral international organizations have identified improving outdoor microclimates and reducing the risks associated with extreme land-surface heat exposure as central priorities in the development of climate-adaptive cities (4). The United Nations and the World Health Organization have also issued health prevention and protection guidelines for extreme heat on multiple occasions (5).

Streets constitute a primary setting for residents’ daily mobility, and the near-surface thermal environment directly influences their outdoor experience. However, conventional urban microclimate studies have largely relied on Land Surface Temperature (LST) to characterize heat island effects at the city or regional scale. Although LST is effective in identifying macro-scale thermal “hotspots,” it provides limited representation of the actual level of land-surface heat exposure experienced by pedestrians at the near-surface scale (6). Therefore, investigating the relationship between micro-scale street-view characteristics and the thermal environment from a pedestrian perspective has become an important direction for fine-grained street planning.

From a technological perspective, the integration of street-view imagery can objectively capture the three-dimensional physical morphology of streets, effectively addressing the limitations of conventional LST data in near-surface observations and providing critical data support for in-depth analysis and optimization of land-surface heat exposure on urban streets (7). With the aid of deep learning algorithms, researchers can automatically and efficiently extract multidimensional street morphology metrics from street-view imagery (8). Existing studies have demonstrated that these three-dimensional metrics significantly influence the microclimate within street environments. In terms of geometric morphology, SVF directly affects the receipt of solar radiation and nocturnal heat dissipation (9). From an ecological perspective, GVI not only provides physical shading but also effectively reduces local temperatures through plant transpiration. By converting complex three-dimensional street environments into quantifiable parameters, these metrics establish a solid foundation for accurately examining the potential associations between micro-scale built environments and land-surface heat exposure.

Although the use of street-view imagery for microclimate assessment has become a major research focus, existing approaches still face two major limitations. First, they have difficulty accurately characterizing spatial differentiation. The complexity of urban environments results in substantial spatial variations in the effects of individual street-view metrics on LST. Conventional statistical models are often constrained by single-scale assumptions, making it difficult to accurately capture such spatial variations (10). Second, nonlinear relationships and threshold effects are often overlooked. The relationships between street-view metrics and temperature are not simply additive and linear but instead exhibit distinct turning points. For example, the cooling benefits of vegetation have an upper limit, while the effects of building enclosure on the thermal environment are not necessarily enhanced with increasing enclosure. Because conventional linear approaches cannot identify these critical thresholds, existing thermal environment optimization strategies have difficulty providing explicit quantitative criteria for fine-grained street renewal (11).

To address the aforementioned gaps, this study takes “street-view big data” in high-density urban areas as its starting point and establishes a research framework of “identifying spatial differences—identifying nonlinear thresholds—implementing refined and classified governance.” The framework consists of four steps. First, street classification: POI data and the K-means clustering algorithm are used to classify streets according to their functions. Second, extraction of key indicators: LST is obtained, and multidimensional street morphology metrics are extracted using deep learning techniques. Third, analysis of potential associations: MGWR and the XGBoost-SHAP model are jointly used to reveal the spatial differences and nonlinear relationships between individual street-view metrics and the thermal environment. Fourth, development of optimization strategies: a “current condition–sensitivity matrix” is constructed by combining regression coefficients and nonlinear thresholds to assess the risk levels of land-surface heat exposure across different types of streets, and refined cooling optimization strategies are proposed for representative streets (Figure 1).

**Figure 1:**
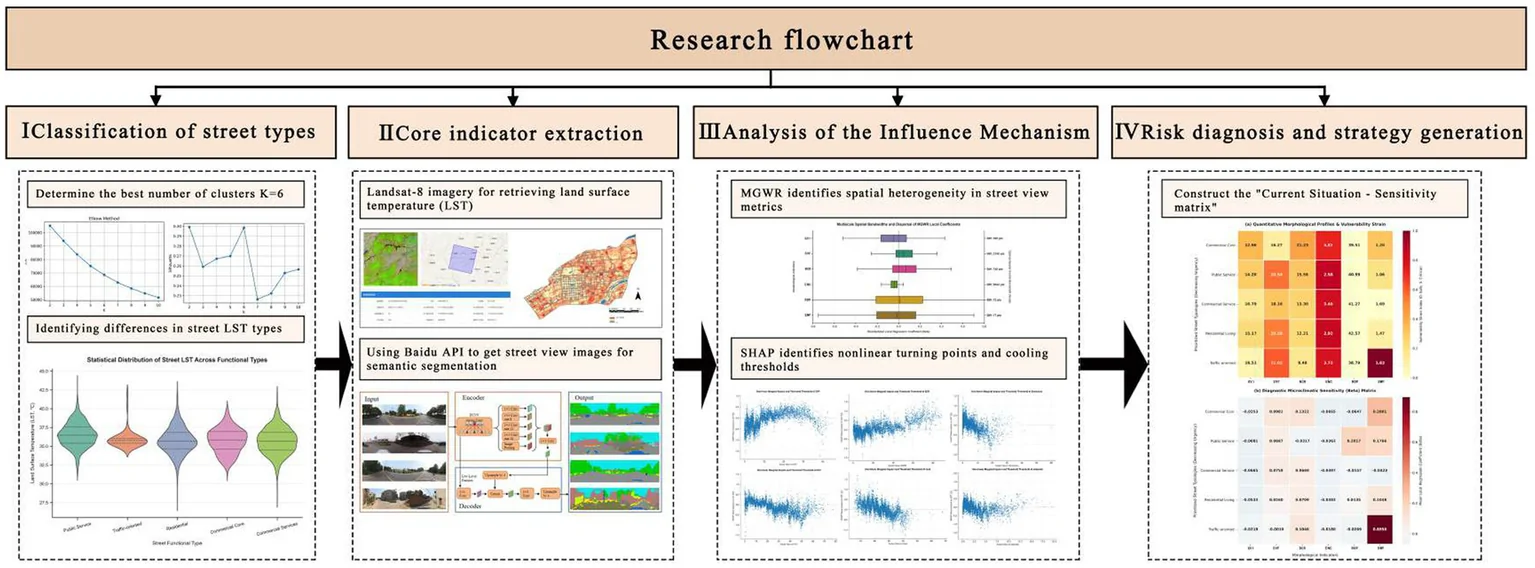
Workflow diagram.

## 2 Data and methods

### 2.1 Research area and data acquisition

Luolong District, Luoyang, Henan Province, is located on the Yiluo River alluvial plain. The terrain forms a dustpan-shaped open basin, with higher elevations in the northwest and lower elevations in the southeast. Located in the transitional zone between the subtropical and warm temperate zones, the district has a continental monsoon climate, with distinct monsoons as its main climatic characteristic. The dustpan-shaped basin is prone to air stagnation during summer, which, combined with the highly dense and artificially hardened underlying surface in the central urban area, amplifies the cumulative effects of land-surface heat exposure. Therefore, the central urban area of Luolong District, Luoyang, Henan Province, was selected as the main study area.

To systematically quantify the built environment at the street level, a fine-grained spatial sampling strategy was adopted. The complete road network within the study area was extracted, and sampling points were generated at strict 50-m intervals along the centerlines of all roads. These sampling points served as precise geographic coordinates for obtaining street-view imagery. This high-resolution sampling approach ensured comprehensive and detailed spatial coverage of the street environment across the study area, providing a solid data foundation for subsequent micro-scale assessment of land-surface heat exposure (Figure 2).

**Figure 2:**
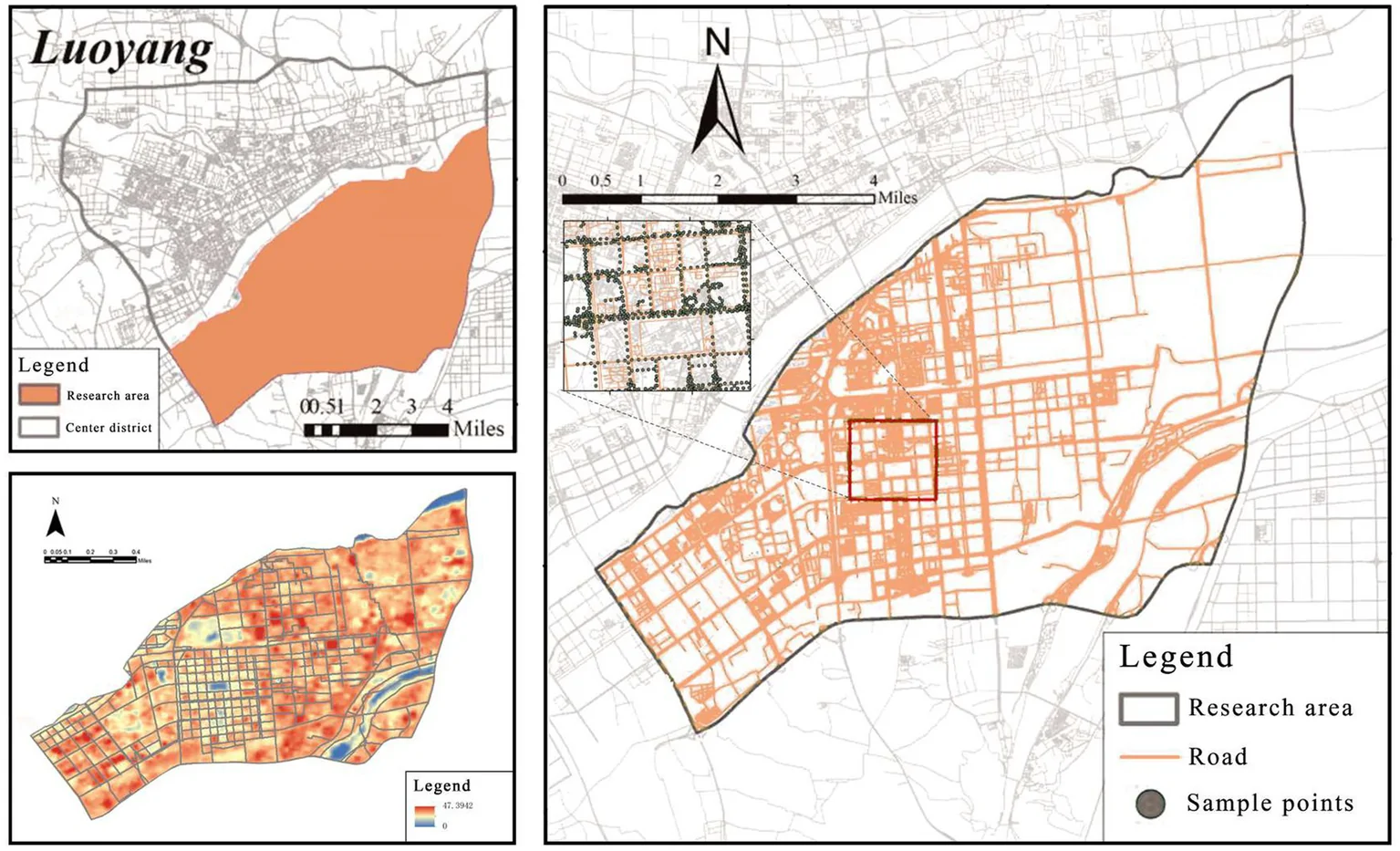
Research location.

For the dependent variable, continuous LST data for Luolong District were obtained (12), as shown in Figure 2, and used as the primary indicator of land-surface heat exposure. For the independent variables, high-density Baidu street-view imagery was obtained, and a pretrained DeepLabv3 + deep learning semantic segmentation architecture was employed to identify and calculate street-view metrics, including GVI, SVF, ENC, pavement grayscale (GrVI), openness (SOI), BCR, RDP, and SWP, within the street-level field of view (13). Finally, GIS Spatial Join was used to accurately match all street-view metric variables to 9,157 street-grid sampling points in Luolong District (14). A 30-m-diameter buffer zone was then established around each sampling point, and LST was spatially linked to the buffer zones to obtain the mean temperature surrounding each sampling point. This mean temperature was used to represent the influence of the street-view metrics at each sampling point on the surrounding land-surface heat exposure, thereby establishing a complete underlying spatial database.

#### 2.1.1 Dependent variable: land surface temperature retrieved from remote sensing imagery

This study uses LST as the dependent variable to measure the level of local land-surface heat exposure in summer (15). The data were obtained from a cloud-free summer remote sensing image acquired by the Landsat 8 satellite on July 30, 2025 (16). The image was acquired during the typical summer high-temperature period in Luoyang, allowing it to adequately represent the extreme land-surface heat exposure conditions in the study area. To eliminate the influence of atmospheric factors (17), this study employed the Single-Channel Algorithm based on the Radiative Transfer Equation (RTE) to retrieve LST from Landsat 8 Band 10 (18). The calculation process consisted of three main steps:

(1) Radiometric calibration and calculation of the atmospheric thermal radiative transfer equation:

The original digital number (DN) values of remote sensing image pixels were converted into sensor spectral radiance values $\;L_{\lambda }$ . The thermal infrared radiative transfer equation can be expressed as follows:


$$L_{\lambda }=\left[\in_{\lambda }B\left(T_{s}\right)+\left(1-\in_{\lambda }\right)L_{↓}\right]\tau +L_{↑}$$

where τ represents the atmospheric transmittance in the thermal infrared band; $L_{↑}$ and $L_{↓}$ denote the upward and downward atmospheric radiative fluxes, respectively.

(2) Accurate estimation of surface emissivity (*ε*) and fractional vegetation cover (FVC):

The mixed pixel decomposition method was adopted to refine the estimation of surface emissivity $\left(\epsilon_{\lambda }\right)$ for street underlying surfaces based on fractional vegetation cover (FVC):


$$\in_{\lambda }=\in_{v}FVC+\in_{s}\left(1-FVC\right)+d\in$$

where $\in_{v}$ and $\in_{s}$ represent the pure pixel emissivity values of vegetation and soil, respectively; d∈ represents the geometric contribution of multiple reflections caused by three-dimensional surface roughness.

The derivation of FVC is based on the Normalized Difference Vegetation Index (NDVI), which is calculated using the near-infrared (Band 5) and red (Band 4) bands of Landsat 8:


$$NDVI=\frac{Band5-Band4}{Band5+Band4}$$

Subsequently, FVC was calculated using the pixel dichotomy model:


$$FVC=\frac{NDVI-NDVI_{soil}}{NDVI_{veg}-NDVI_{soil}}$$

where $NDVI_{soil}$ and $NDVI_{veg}$ represent the NDVI values for areas with complete bare-soil cover and complete vegetation cover, respectively. Based on the actual conditions of the study area, the NDVI values corresponding to the 5 and 95% percentiles of the cumulative probability distribution were used as $NDVI_{soil}$ and $NDVI_{veg}$ , respectively.

(3) Retrieval of actual surface temperature using the inverse Planck function:

Based on the blackbody radiance retrieved at the sensor level, $B\left(T_{s}\right)$ , the actual land surface temperature $T_{s}$ was calculated by applying the inverse Planck function. The unit of $T_{s}$ is Kelvin (K), which was subsequently converted to degrees Celsius (°C):


$$T_{s}=\frac{K_{2}}{\ln\left(\frac{K_{1}}{B\left(T_{s}\right)}+1\right)}$$

For Landsat-8 Thermal Infrared Sensor (TIRS) Band 10, the radiometric calibration constants are fixed as follows: $K_{1}=\frac{774.89W}{m^{2}\cdot \mathrm{sr}\cdot \mu m}$ , $K_{2}=1321.08K$ .

#### 2.1.2 Independent variables: semantic segmentation-based metrics derived from Baidu street-view imagery

To comprehensively characterize the potential associations between urban street canyon morphology, the underlying surface of the built-up area, and the thermal environment, this study constructed independent variables representing two types of spatial morphology.

Using the road network of Luolong District as the basis, 9,157 sampling points were set at 50-m intervals. From July 26 to August 7, 2025, street-view images in four directions at each sampling point were obtained through the Baidu Maps API and stitched together into panoramic images to ensure temporal consistency between the street-view morphology and LST data. To convert the images into quantifiable spatial metrics, a DeepLabv3 + deep learning model pretrained on the Cityscapes dataset was used to perform semantic segmentation of the street-view images (19). By accurately identifying the proportions of elements such as vegetation, sky, buildings, motor vehicle lanes, and sidewalks in the images, the following core metrics were calculated:

(1) Three-dimensional street-view metrics

*GVI*: The proportion of green vegetation pixels (e.g., tree canopies and shrubs) within the pedestrian visual field relative to the total pixels of the panoramic image. This metric not only reflects the visual greenness level but also characterizes the shading capacity of vegetation canopies in blocking incoming shortwave solar radiation (20).

*SVF*: The proportion of visible sky above a sampling point that is unobstructed by buildings or vegetation canopies. SVF significantly influences the amount of solar radiation received by streets during daytime and the efficiency of nocturnal heat dissipation (21).

*ENC*: The proportion of building pixels on both sides of the street within the visual field relative to the total pixels of the panoramic image. This metric is commonly used to quantify the degree of street canyon enclosure and its influence on local heat accumulation (22).

*SOI*: The proportion of spaces with high visual permeability and extended sight distance within the visual field, reflecting the overall openness of streets at the human scale.

*SWP*: The proportion of pedestrian-accessible paved surface pixels within street-view imagery (21).

(2) Planar street-view metrics

*GrVI*: The proportion of artificial gray infrastructure, including impervious pavement and masonry materials, within the visual field relative to the total pixels of the panoramic image.

*RDP*: The proportion of motorized road area within a buffer zone centered on the sampling point, with a radius of R (15 m in this study), relative to the total area of the buffer zone.

*BCR*: The proportion of building footprint area relative to the total area of the same spatial buffer zone.

### 2.2 Data preprocessing

To ensure the accuracy and robustness of the spatial regression analysis and machine learning models, a series of preprocessing and screening procedures were conducted on the extracted raw dataset, including missing value and erroneous value cleaning, non-parametric tests, collinearity analysis, and correlation analysis.

#### 2.2.1 Data cleaning

First, the dataset was preprocessed by removing missing and erroneous values. After data cleaning, the 9,171 street sampling points across the study area achieved a 0.00% missing rate (Table 1), ensuring the integrity of the continuous spatial analysis. Descriptive statistics of the indicators further revealed that the street-view metric data in the central urban area of Luolong District exhibited non-normal distributions.

**Table 1:** Descriptive statistics of indicators.

Variable	*N*	Mean	Std	Min	Median	Max	*p*-value
LST (°C)	9,171	35.825	1.762	27.067	35.769	43.638	< 0.001
GVI (%)	9,171	15.476	17.259	0.000	5.608	77.602	< 0.001
SVF	9,171	19.499	12.328	0.000	23.453	44.675	< 0.001
BCR (%)	9,171	13.300	19.178	0.000	3.780	88.313	< 0.001
ENC	9,171	3.220	4.316	0.000	1.100	60.000	< 0.001
GrVI	9,171	16.171	21.347	0.000	6.698	92.84	< 0.001
SOI	9,171	65.227	31.325	5.255	60.645	98.65	< 0.001
RDP (%)	9,171	41.444	19.957	0.000	42.138	98.650	< 0.001
SWP (%)	9,171	1.458	4.312	0.000	0.000	73.125	< 0.001

#### 2.2.2 Kruskal–Wallis non-parametric test

Given that the aforementioned street-view metrics and land-surface heat exposure data exhibited significant skewness and non-normal distributions across different spatial contexts, conventional parametric analyses based on assumptions of normality and homogeneity of variance, such as Analysis of Variance (ANOVA), were no longer applicable. Therefore, this study directly employed the Kruskal–Wallis non-parametric test, using the mean rank of each variable group as the core statistic. Without relying on assumptions of variance homogeneity or normal distribution, this method robustly identifies significant differences in environmental characteristics among different street types (Table 2).

**Table 2:** Non parametric testing of street view indicators.

Variable	H	P_value
LST	219.2567	0
GVI	33.1523	0.000004
SVF	85.0424	0
BCR	32.1012	0.000006
GrVI	28.0902	0.000035
SOI	50.767	0
ENC	43.1043	0
RDP	37.1523	0.000001
SWP	33.148	0.000004

The results showed that the H statistic of LST reached 219.2567, with *p* = 0.000. The eight street-view independent variables also exhibited statistically significant differences at the level of *p* < 0.001. These findings demonstrate that both street-level land-surface heat exposure and street-view metrics in the central urban area of Luolong District exhibit highly significant spatial heterogeneity among streets with different functional types. Therefore, conducting a global analysis without distinguishing street functions would overlook this functional differentiation mechanism. Accordingly, the K-means clustering method was introduced in the subsequent analysis to classify streets in the central urban area of Luolong District according to their functional characteristics.

#### 2.2.3 Collinearity and correlation analysis

In high-density built environments, the indicators often exhibit strong physical dependence and conceptual overlap, resulting in severe multicollinearity in spatial statistics. Directly inputting these highly correlated and redundant independent variables into MGWR or machine learning models would increase the errors of spatial regression coefficients and reduce the accuracy of machine learning models. Therefore, this study introduced the Pearson Correlation Coefficient and Variance Inflation Factor (VIF) for dual correlation diagnostics. As shown by the global Pearson correlation matrix, the linear correlation coefficient between BCR and GrVI was as high as 0.98, while those between SVF and SOI and between RDP and SOI reached 0.937 and 0.963, respectively (Figure 3). The corresponding initial VIF diagnostics indicated that some variables exceeded the conventional threshold. To ensure the statistical rigor of model estimation and the stability of feature attribution, this study systematically removed highly overlapping and redundant indicators. The collinearity analysis showed that, except for SWP, all other indicators exceeded the critical threshold for multicollinearity (VIF > 10). However, GVI, SVF, and RDP are indispensable independent variables in urban microclimate research (23). After removing SOI, GrVI, and BCR, whose definitions substantially overlap with those of other indicators, the collinearity values of the remaining indicators decreased to below 5, indicating that there was no longer a serious variance inflation problem caused by multicollinearity in the multiple regression and machine learning models. The remaining indicators were therefore retained (Table 3).

**Figure 3:**
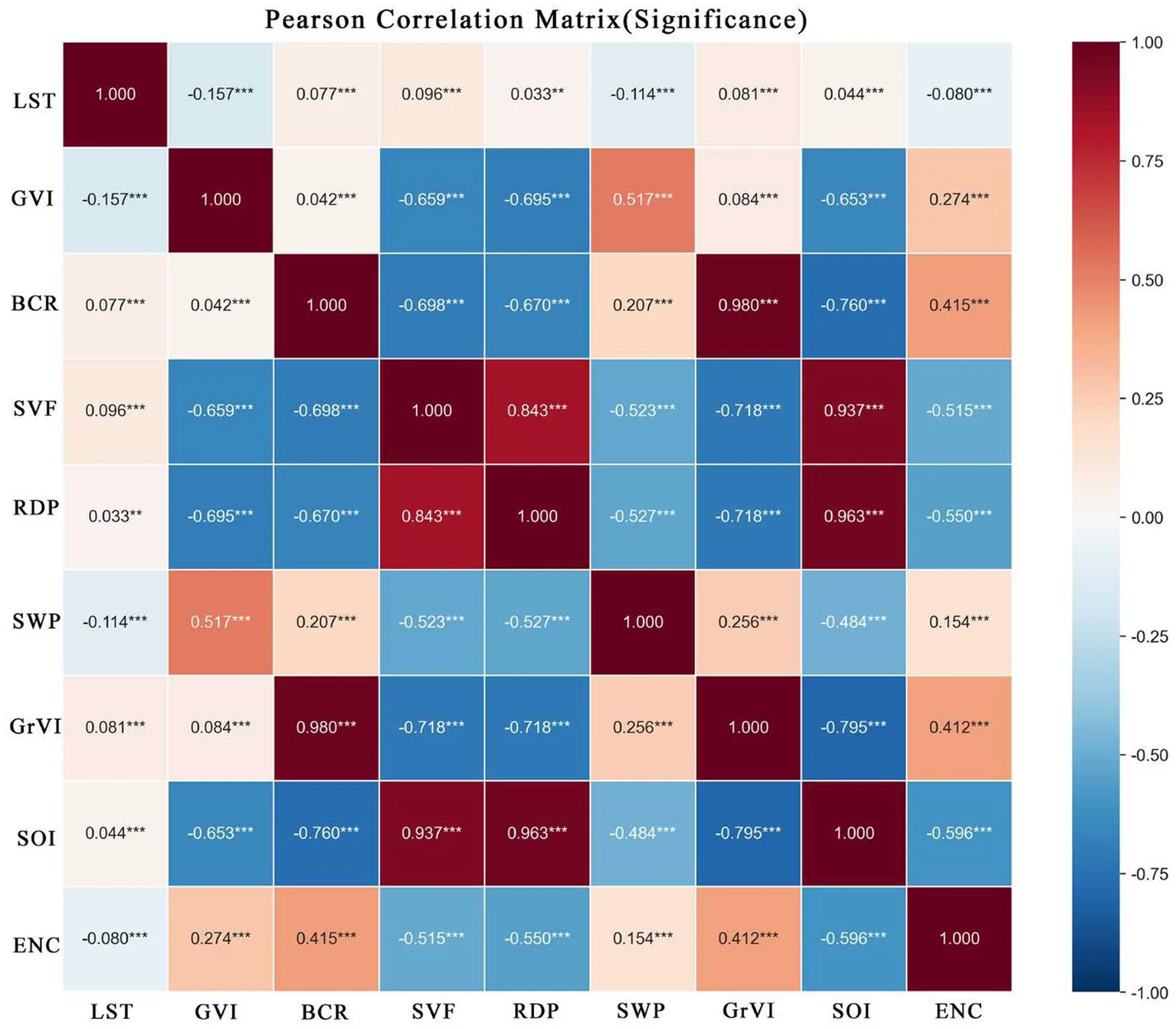
Correlation analysis chart of street view indicators.

**Table 3:** Collinearity analysis of street view indicators.

Excluding collinearity before exclusion			Decision	Excluding collinearity		
Variable	VIF	Level		Variable	VIF	Level
GVI	19374742.67	Serious	Reserved (representing three-dimensional vegetation ecological cooling)	GVI	2.17	Good
ENC	1731266.867	Serious	Reserved	ENC	1.568	Good
RDP	46.378	Serious	Reserved (representing two-dimensional asphalt pavement physical heat storage)	RDP	4.432	Good
SVF	26.001	Serious	Reserved (representing three-dimensional long short wave radiation interception)	SVF	3.77	Good
SWP	1.972	Good	Reserved	SWP	1.549	Good
BCR	29.433	Serious	Elimination (overlaps with other indicator concepts)			
SOI	63821688.96	Serious	Elimination (showing strong linear overlap with SVF and RDP)			
GrVI	29640032.73	Serious	Elimination (showing strong linear overlap with BCR)			

### 2.3 Multiscale geographically weighted regression

To accurately capture local spatial heterogeneity while scientifically interpreting the effects of different street-view metrics on LST, this study introduced the MGWR model.

MGWR overcomes the limitation of a single fixed bandwidth in traditional regression models by allowing each street-view metric as an independent variable to possess its own spatially varying bandwidth. The model can be expressed as follows:


$$Y_{i}=\beta_{0}\left(u_{i},v_{i}\right)+\overset{k}{\underset{j=1}{\sum }}\beta_{bwj}\left(u_{i},v_{i}\right)X_{ij}+\varepsilon_{i}$$

where $Y_{i}$ represents the dependent variable, LST, at grid sampling point i; $\left(u_{i},v_{i}\right)$ denotes the two-dimensional geographic centroid coordinates of sampling point i; $\beta_{0}\left(u_{i},v_{i}\right)$ represents the spatial intercept term; $X_{ij}$ denotes the observed value of the jth street-view metric at sampling point i; and $\varepsilon_{i}$ represents the random error term. $\beta_{bwj}\left(u_{i},v_{i}\right)$ is the regression coefficient, where the subscript bwj indicates the independent optimal bandwidth specifically determined for the j independent variable when estimating local spatial heterogeneity.

Regarding the selection of the spatial weighting kernel function, this study adopted the adaptive Gaussian kernel function, which is widely recognized as a general-purpose approach in spatial analysis, to dynamically define the spatial weights between pairs of locations (24):


$$w_{ij}=\exp\left(-\frac{1}{2}{\left(\frac{d_{ij}}{b_{i}}\right)}^{2}\right)$$

where $d_{ij}$ represents the straight-line Euclidean distance between sampling point i and its neighboring point j ; $b_{i}$ denotes the adaptive distance threshold dynamically determined through the backfilling algorithm. This study employed the Akaike Information Criterion corrected (AICc) as the evaluation criterion for selecting the globally optimal bandwidth. By conducting MGWR calculations for all 9,157 sampling points across the study area, the model generated a set of bandwidth values for each street-view metric to characterize its corresponding spatial scale of influence, thereby revealing the local spatial heterogeneity of different variables across geographic locations.

To verify the scientific validity and necessity of selecting the MGWR model, this study compared the performance of the traditional Ordinary Least Squares (OLS), Geographically Weighted Regression (GWR), and MGWR models (Table 4). The results showed that, compared with the global OLS model, the spatial regression models achieved a significant improvement in fitting accuracy. Further comparison between GWR and MGWR showed that although the traditional GWR had a higher R^2^, it had a risk of local overfitting. In contrast, MGWR effectively avoided overfitting through its adaptive capability of using multi-scale bandwidths. Its Adjusted R^2^ (Adjusted R^2^ = 0.6001) was not only higher than that of GWR, but its AICc (AICc = 4168.8766) also decreased substantially. The substantial reduction in AICc, together with the increase in Adjusted R^2^, demonstrates that MGWR can achieve both high fitting accuracy and low information loss when handling high-dimensional and complex urban street-view environment variables. Therefore, there is sufficient statistical evidence for selecting MGWR to investigate spatial heterogeneity in this study.

**Table 4:** Performance comparison of OLS, GWR, and MGWR models.

Model	R^2^	Adjusted R^2^	RSS	AICc
OLS	0.0816	0.0788	1836.8169	5519.5843
GWR	0.6737	0.5861	652.6862	4512.0695
MGWR	0.6527	0.6001	694.5149	4168.8766

### 2.4 Machine learning regression model

Building upon the spatial scale diagnosis provided by MGWR, machine learning algorithms were further introduced to capture the nonlinear thresholds, turning points, and interaction effects exhibited by built environment–microclimate relationships under complex interventions.

Given the “black-box” nature of multidimensional nested decision trees, the nonlinear potential associations and marginal turning points between individual street-view metrics and land-surface heat exposure are difficult to interpret directly (25). To address this limitation, the SHAP (SHapley Additive exPlanations) interpretability model, based on a unified game-theoretic framework, was introduced (26). The core advantage of the SHAP framework is that, by calculating Shapley values, it can decompose the model’s predicted LST for each street sampling point into the sum of the marginal contributions of individual morphological independent variables. The underlying logic of the SHAP model is to treat the input morphological features of each sampling point as a cooperative game, which can be expressed as:


$$g\left(z^{\prime }\right)=\phi_{0}+\overset{M}{\underset{i=1}{\sum }}\phi_{i}z\prime_{i}$$

where $g\left(z^{\prime }\right)$ represents the local explanation model; $z^{\prime }\in {\left\{0,1\right\}}^{M}$ denotes the binary variable indicating whether a feature is included in the coalition subset of the game (where M represents the total number of independent variables, and M = 6 in this study); $\phi_{0}$ represents the global mean prediction of urban thermal conditions when no independent variables are included in the model; and $\phi_{i}$ represents the actual SHAP contribution value (Shapley Value) of the i urban morphological factor to local Land Surface Temperature (LST).

For each specific street-level independent variable i , its core contribution value $\phi_{i}$ is strictly calculated as the weighted average of its conditional expected marginal contributions across all possible subsets of variable combinations. The original calculation formula is as follows:


$$\phi_{i}=\underset{S\subseteq F\setminus \left\{i\right\}}{\sum }\frac{\left|S\right|!\left(M-\left|S\right|-1\right)!}{M!}\left[f_{x}\left(S\cup \left\{i\right\}\right)-f_{x}\left(S\right)\right]$$

where F represents the full set of feature variables; S denotes any sampled subset of independent variables after excluding the currently evaluated factor i; |S|! represents the feature space dimensionality contained within subset S; M! represents the total number of permutations of all features; $f_{x}\left(S\cup \left\{i\right\}\right)$ and $f_{x}\left(S\right)$ represent the joint conditional prediction outputs of the machine learning model for local LST when factor i is included and excluded, respectively. If $\phi_{i}>0$ , the corresponding street-view metric contributes to warming effects under the current spatial condition; if $\phi_{i}<0$ , the street-view metric exhibits a significant cooling effect.

Different machine learning architectures exhibit substantial differences in fitting accuracy, generalization capability, and noise robustness when processing high-dimensional spatial morphological data. To evaluate the predictive performance of the models for street-view metrics and compare them with conventional linear methods, five machine learning and deep learning models were selected: (1) Ordinary Least Squares regression (OLS) (26); (2) Ridge Regression (27); (3) LightGBM (28); (4) XGBoost (25); and (5) CatBoost (29).

The dataset was randomly divided into training and testing subsets, with 80% of the samples used for model training and 20% reserved for model testing. To ensure the robustness of model evaluation, five-fold cross-validation was adopted. Three statistical metrics, including the coefficient of determination (R^2^), Root Mean Square Error (RMSE), and Mean Absolute Error (MAE), were employed to evaluate and compare the performance of each model. The final evaluation results are presented in Figure 4.

**Figure 4:**
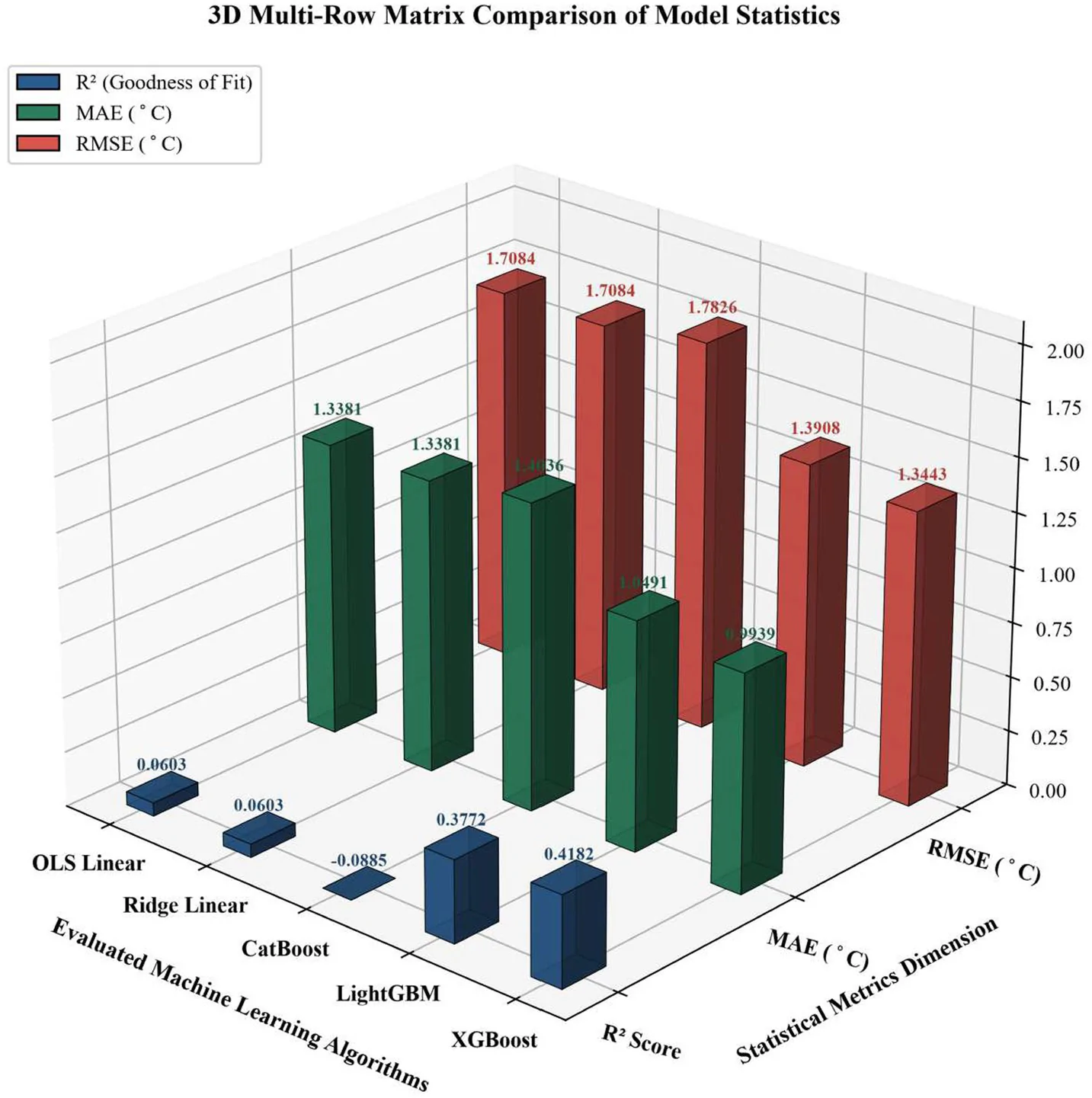
Comparison diagram of 3D matrix of models.

Among all evaluated models, the XGBoost algorithm based on the boosting framework demonstrated the strongest capability for global data representation and generalization. It achieved the highest overall R^2^ value of 0.4182 and the lowest prediction errors (RMSE = 1.3443 °C, MAE = 0.9939 °C). The explanatory performance of XGBoost for LST substantially outperformed that of traditional linear regression models. When processing fine-scale street-view features, XGBoost was able to more effectively reproduce the nonlinear relationships between street-view characteristics and land-surface heat exposure observed in real-world environments (21).

Benefiting from its second-order Taylor expansion-based optimization and continuous gradient boosting mechanism, XGBoost can be effectively integrated with the Tree-SHAP interpretability framework to provide highly robust analyses of Marginal Threshold Profile and stable convergence of turning points (22). Therefore, XGBoost was ultimately selected as the modeling framework for subsequent global SHAP interpretation and nonlinear threshold identification.

To evaluate the effectiveness of the model in complex urban environments, this study conducted a spatial autocorrelation test on the prediction residuals of XGBoost. The results showed that the global Moran’s I was 0.7098 (*p* < 0.001, Z-score = 143.27). This indicates that, because local meteorological conditions and anthropogenic heat emissions were not included in the model, the prediction residuals exhibited significant spatial autocorrelation. Despite the presence of spatial autocorrelation in the residuals, the nonlinear cooling thresholds extracted in this study remain highly reliable, mainly for the following reasons:

(1) SHAP separates marginal contributions: The SHAP method can calculate the independent “marginal contribution” of a single indicator to temperature. It eliminates the interference caused by differences in absolute temperature among different locations, making the identified nonlinear patterns of shading and ventilation more statistically stable.
(2) Complementary advantages of the two models: The spatial autocorrelation of the residuals further demonstrates the necessity of introducing the MGWR model, as discussed above. By combining “MGWR (addressing spatial heterogeneity) + XGBoost (extracting nonlinear thresholds),” this study effectively compensates for the limitations of a single model.
(3) Clustering controls spatial interference: K-means street functional clustering was performed before extracting the thresholds, as described below. This step grouped streets with similar anthropogenic heat sources and building characteristics into the same group, effectively reducing the interference of global spatial heterogeneity with the analysis results.

### 2.5 K-means clustering algorithm

To avoid the shortcomings of a “one-size-fits-all” planning approach in microclimate-oriented urban renewal, the street network within the study area was first classified according to functional types. Streets with different functional characteristics exhibit fundamental differences in anthropogenic heat emissions, pavement proportions, pedestrian activity intensity, and other attributes. Therefore, Point of Interest (POI) data were introduced, and the K-means clustering algorithm was employed to classify urban street types (30).

#### 2.5.1 POI data extraction

The study was based on sampling points along the GIS road centerlines, with a 30-m geospatial buffer zone constructed around each sampling point as the basic statistical unit for socioeconomic functions. POI data for the study area were obtained through the Baidu Maps API on August 7, 2025. Fifteen original core POI categories were used to characterize street functions. These 15 categories specifically included: transportation facilities and road-related facilities; catering, shopping services, daily-life services, and indoor facilities; commercial and residential properties and accommodation services; companies and enterprises; government agencies and social organizations, educational and cultural services, healthcare services, and public facilities; and scenic spots and sports and leisure services.

#### 2.5.2 Determination of the optimal K value for the K-means clustering algorithm

K-means is a classical unsupervised machine learning algorithm. Its core principle is to iteratively calculate the feature distances between sample points and cluster centroids, automatically grouping data with similar attributes into K mutually exclusive clusters. The algorithm ultimately aims to minimize within-cluster variation and maximize between-cluster differences. In this study, Euclidean Distance was employed as the dissimilarity measure. The core optimization objective function, namely the sum of squared errors (SSE), is expressed as follows:


$$SSE=\overset{K}{\underset{k=1}{\sum }}\underset{X_{j}\in C_{k}}{\sum }X_{j}-\mu_{k}^{2}$$

where K represents the predefined number of clusters; $C_{k}$ denotes the set of street functional categories belonging to the kth cluster; $X_{j}$ represents the multidimensional POI functional feature vector of the j sampling point; and $\mu_{k}$ represents the geometric centroid vector of the kth cluster.

Urban POI datasets contain large volumes of complex information on urban activities and business types. Traditional classification approaches that rely on expert knowledge and land-use attributes are inefficient and susceptible to subjective judgments. The K-means algorithm demonstrates strong robustness and computational efficiency when processing large-scale, high-dimensional, and unlabeled urban spatial datasets.

## 3 Results

### 3.1 Street type classification and land-surface heat exposure distribution

To determine the most appropriate number of clusters (K), the elbow method (Figure 5) and the silhouette coefficient method (Figure 6) were employed. As the value of K gradually increased from 2 to 10, the decreasing slope of the SSE curve exhibited the most pronounced critical turning point at K = 6. Meanwhile, the average silhouette coefficient reached its local maximum at this point, indicating that dividing the study area into six street types achieved the optimal balance between high within-cluster cohesion and strong between-cluster separation.

**Figure 5:**
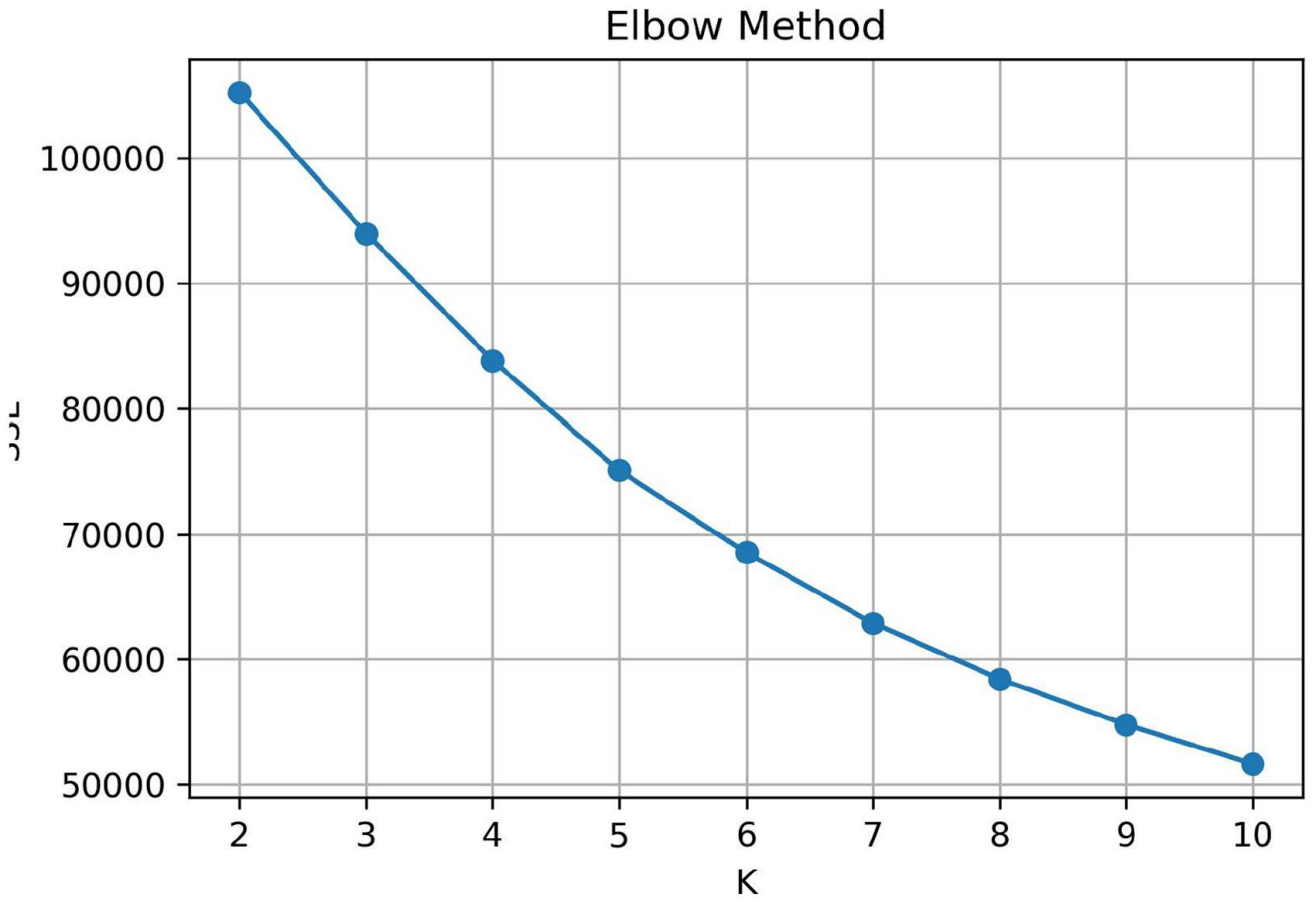
Elbow diagram.

**Figure 6:**
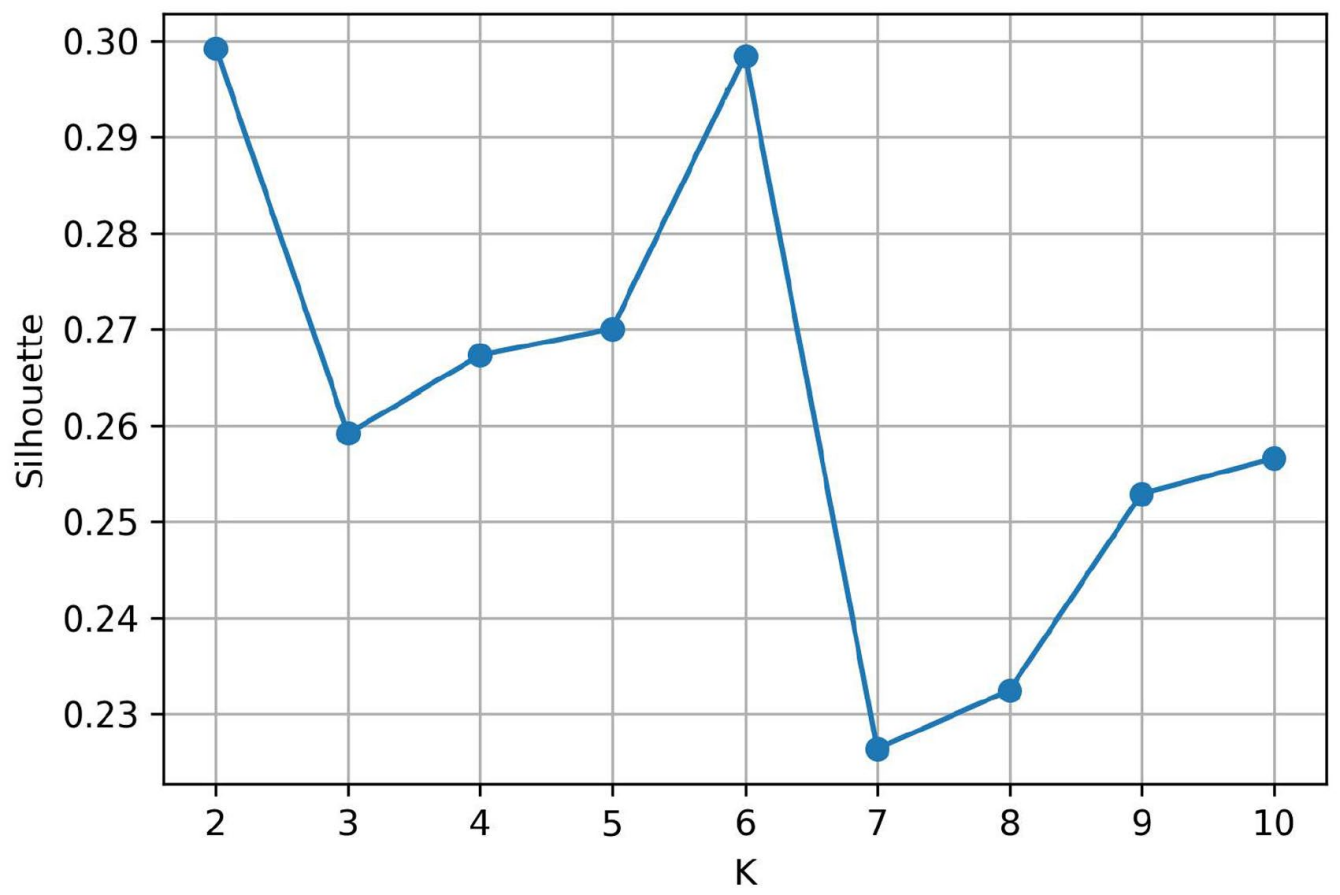
Contour coefficient diagram.

The K-means clustering algorithm identified six distinct functional street types in Luolong District. The mean values of street morphological characteristics and land-surface heat exposure levels exhibited significant functional differentiation among these street categories (Figure 7). To scientifically interpret the urban functional attributes represented by each cluster, the mean values of the clustering centers for 15 POI categories within each street type were calculated. Accordingly, the six street types were formally named as follows (Table 5):

- Cluster 0: public service streets

**Figure 7:**
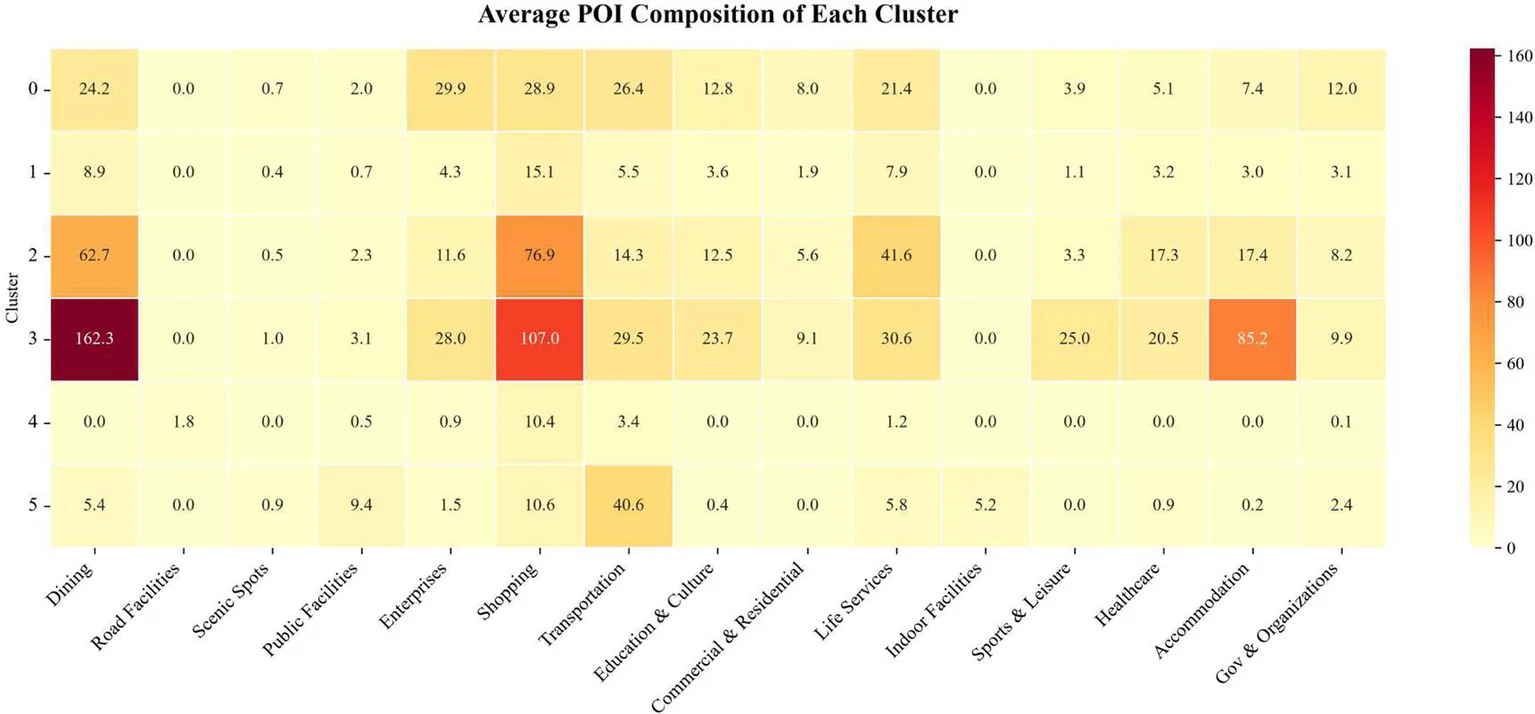
Composition of POIs for each cluster.

**Table 5:** Land-surface heat exposure of different street types.

Street type	*N*	LST	Δ LST	Comprehensive explanation
Public Service	1,161	36.511 °C	+0.685 °C	Severe land-surface heat exposure
Special Function	14	35.971 °C	+0.146 °C	Very small sample size (regression excluded)
Transit-Oriented	45	35.956 °C	+0.131 °C	Moderate land-surface heat exposure
Residential and Neighborhood	4,697	35.756 °C	−0.069 °C	Mild land-surface heat exposure
Commercial Center Core	356	35.715 °C	−0.110 °C	Mild land-surface heat exposure
Commercial Service	2,898	35.674 °C	−0.151 °C	Minimum land-surface heat exposure

This type exhibited relatively high density scores for companies and enterprises (29.86), shopping services (28.86), and transportation facilities (26.39), accompanied by a significant concentration of cultural and educational services as well as government agencies and social organizations.

- Cluster 1: residential and neighborhood streets

The densities of various urban functions within this cluster were generally at medium-to-low levels across the study area. Shopping services (15.09), catering services (8.93), and daily-life services (7.92) constituted the major components of its internal functional structure.

- Cluster 2: commercial service streets

These streets demonstrated a clear concentration of commercial activities, with shopping services (76.93) and catering services (62.71) exceeding those observed in the first two street categories. Meanwhile, daily-life services (41.65) provided important supporting functions.

- Cluster 3: commercial center core streets

This category exhibited extremely high functional density, with the mean density of catering services reaching 162.29 and shopping services reaching 107.03. Accommodation services (85.18) and sports/leisure services (24.97) also reached their highest levels among all street categories.

- Cluster 4: special function streets

This category contained a very limited number of POI facilities overall, but exhibited the highest mean density of road-associated facilities (1.79). Other major components included basic shopping services (10.43) and transportation facilities (3.36).

- Cluster 5: transit-oriented streets

Transportation facilities (40.58) represented the dominant functional component, while public facilities (9.36) and indoor facilities (5.22) also showed relatively high densities. Residential functions and commercial residential functions were absent in this cluster.

Among these categories, Special Function Streets contained only 14 samples, which may easily induce local matrix singularity. Therefore, this category was excluded from subsequent MGWR regression analysis.

To intuitively demonstrate the spatial differences and distribution characteristics of thermal environments among different street types, this study constructed violin plots of current land-surface heat exposure conditions based on the LST values of all sampling points (Figure 8). The specific findings are as follows:

- Public service streets:

**Figure 8:**
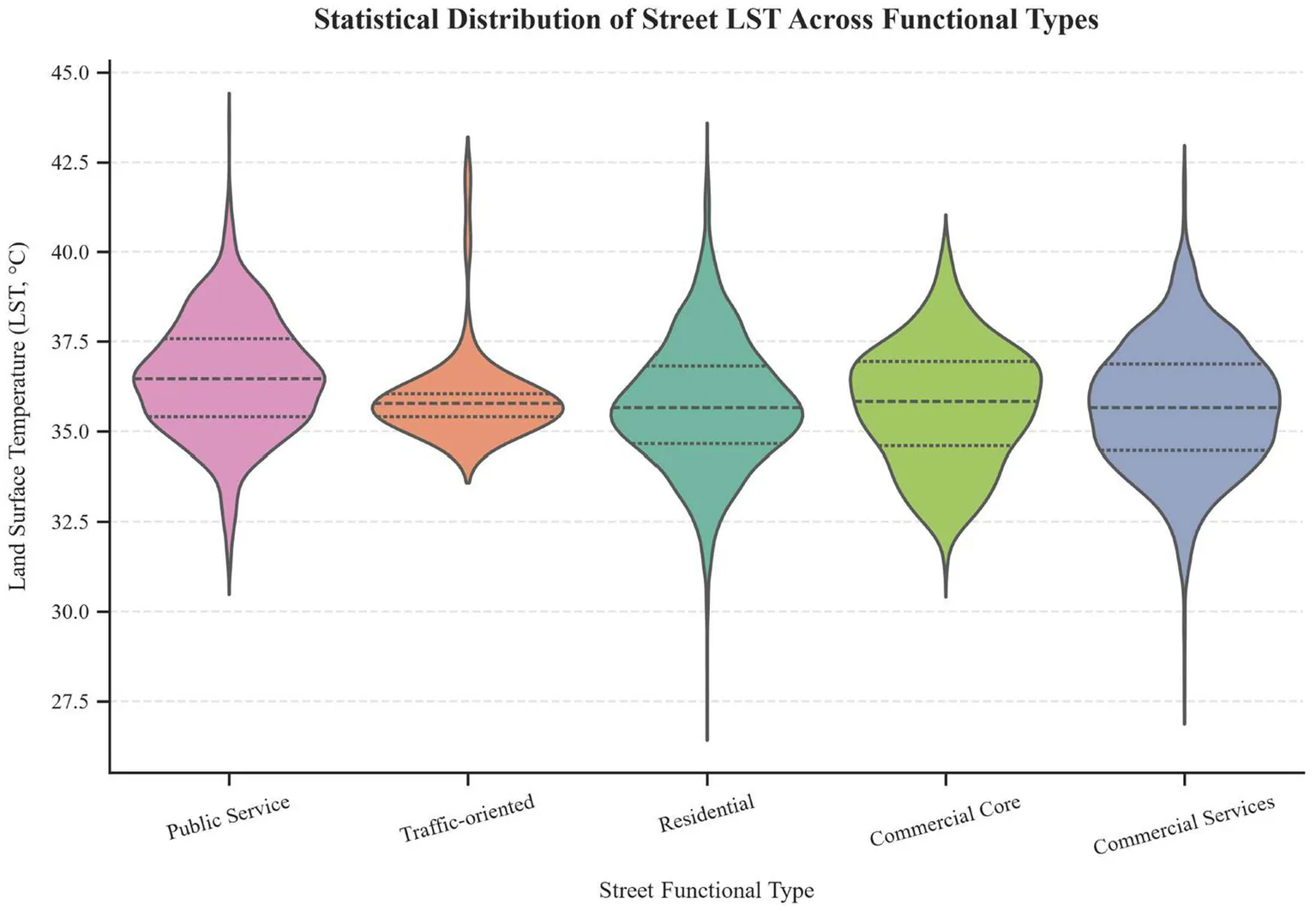
Statistics of land-surface heat exposure in different types of streets.

These streets exhibited relatively high overall temperature distributions. Their median values and interquartile ranges were the highest among all street categories, indicating that they faced the most pronounced land-surface heat exposure risks.

- Residential and neighborhood streets and commercial service streets:

The temperature distributions of these two street types showed relatively long lower-value tails. This suggests the presence of a considerable number of localized cool islands within these streets, potentially resulting from higher vegetation coverage and associated local cooling benefits.

- Transit-oriented streets:

This category exhibited the most concentrated temperature distribution, with the smallest temperature variation range, indicating a high degree of spatial consistency in its internal microclimate and relatively uniform heat distribution.

- Commercial center core streets:

These streets demonstrated a relatively symmetrical and convergent temperature distribution, with an overall homogeneous state of moderate land-surface heat exposure.

### 3.2 Spatially heterogeneous effects of street-view metrics on land-surface heat exposure

To characterize the spatial heterogeneity of the driving effects of street-view morphology on LST and to scientifically identify the geographic spatial bandwidths at which different street-view metrics exert their microclimatic effects, the MGWR model was applied. By conducting a local parameter spatial scan across 9,157 sampling points, MGWR effectively disentangled the spatial scale heterogeneity of different influencing factors.

#### 3.2.1 Multiscale spatial bandwidth analysis of morphological factors

The MGWR model generated independent geographic bandwidths for each street-view metric, directly reflecting the spatial scale at which each metric operates (Table 6). The results indicate that the bandwidths of RDP and SWP are extremely small, corresponding to the micro-scale level. In contrast, GVI exhibit bandwidths at the meso-scale level. The geographic bandwidths of SVF and ENC extend to the macro-scale level.

**Table 6:** Spatial bandwidth of street view indicators.

Street-view metrics	Bandwidths	Spatial scale	Box plot features	Physical mechanism attribution
RDP	12	microscale	Widest box	The thermal environment of pavement is extremely locally sensitive.
SWP	17	microscale	Widest box	Microscopic walking grey sidewalks are highly susceptible to local violent fluctuations caused by building projections and local turbulence.
GVI	389	Mesoscale scale	Medium width box	The 3D visual greening cooling has a stable continuous spillover effect on the neighborhood, and the cooling effect is stable.
SVF	2,198	Macroscopic scale	Narrowest box	The induction of solar radiation land-surface heat exposure by the physical opening width above the city is highly stable on a macro scale.
ENC	5,044	Macroscopic scale	Narrowest box	The three-dimensional street valley boundary exhibits global consistency in the systematic control of local airflow obstruction and nighttime heat flux.

#### 3.2.2 Spatial heterogeneity and distribution characteristics of local regression coefficients of street-view metrics

To further investigate the spatial heterogeneity and distribution characteristics of the effects of street-view metrics on LST, the MGWR local coefficient distributions in relation to multiscale spatial bandwidths (Figure 9) and the spatial pattern maps of local coefficients for street-view metrics (Figure 10) are jointly interpreted as follows:

**Figure 9:**
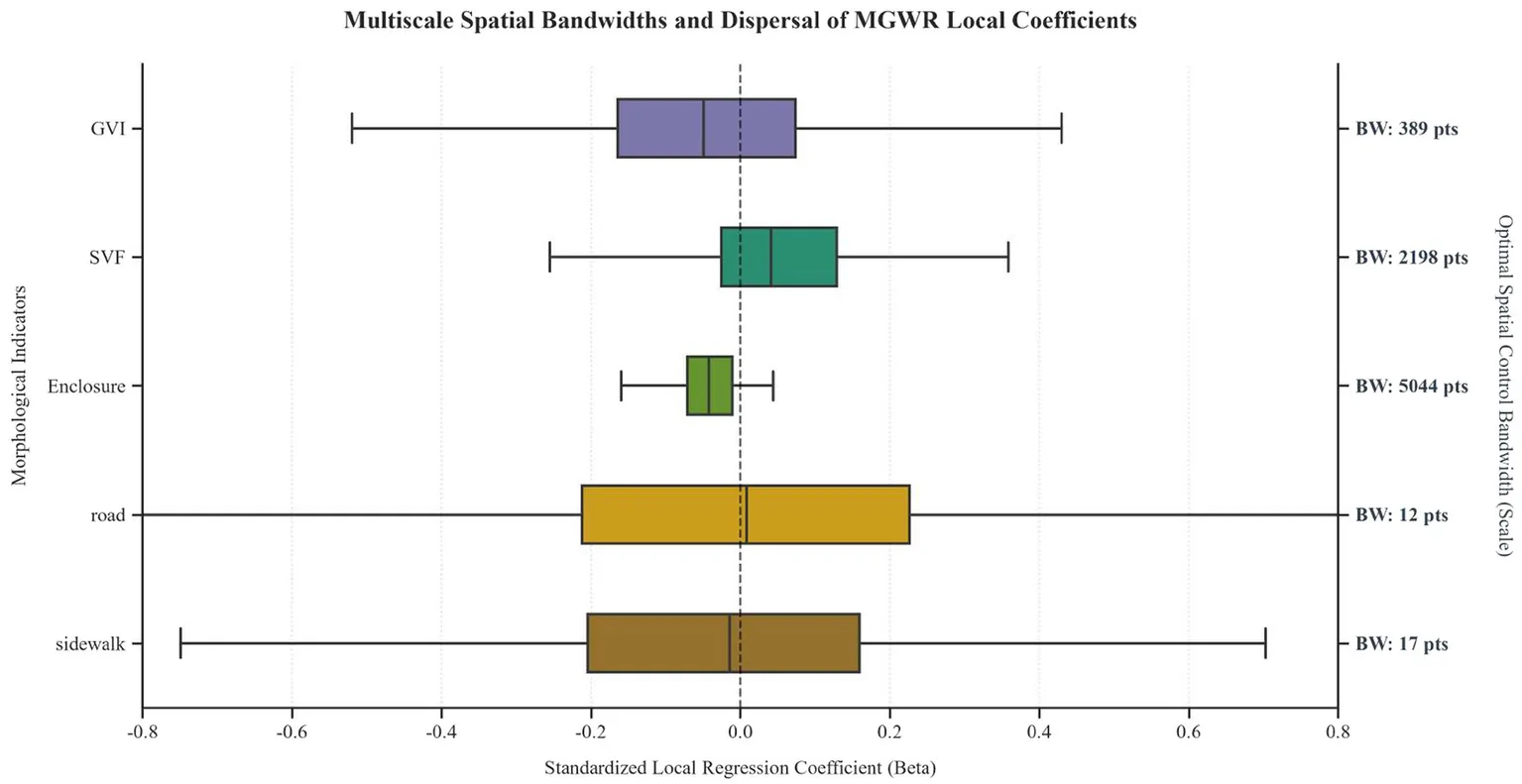
Multi scale spatial bandwidth and local coefficient distribution characteristics of MGWR.

**Figure 10:**
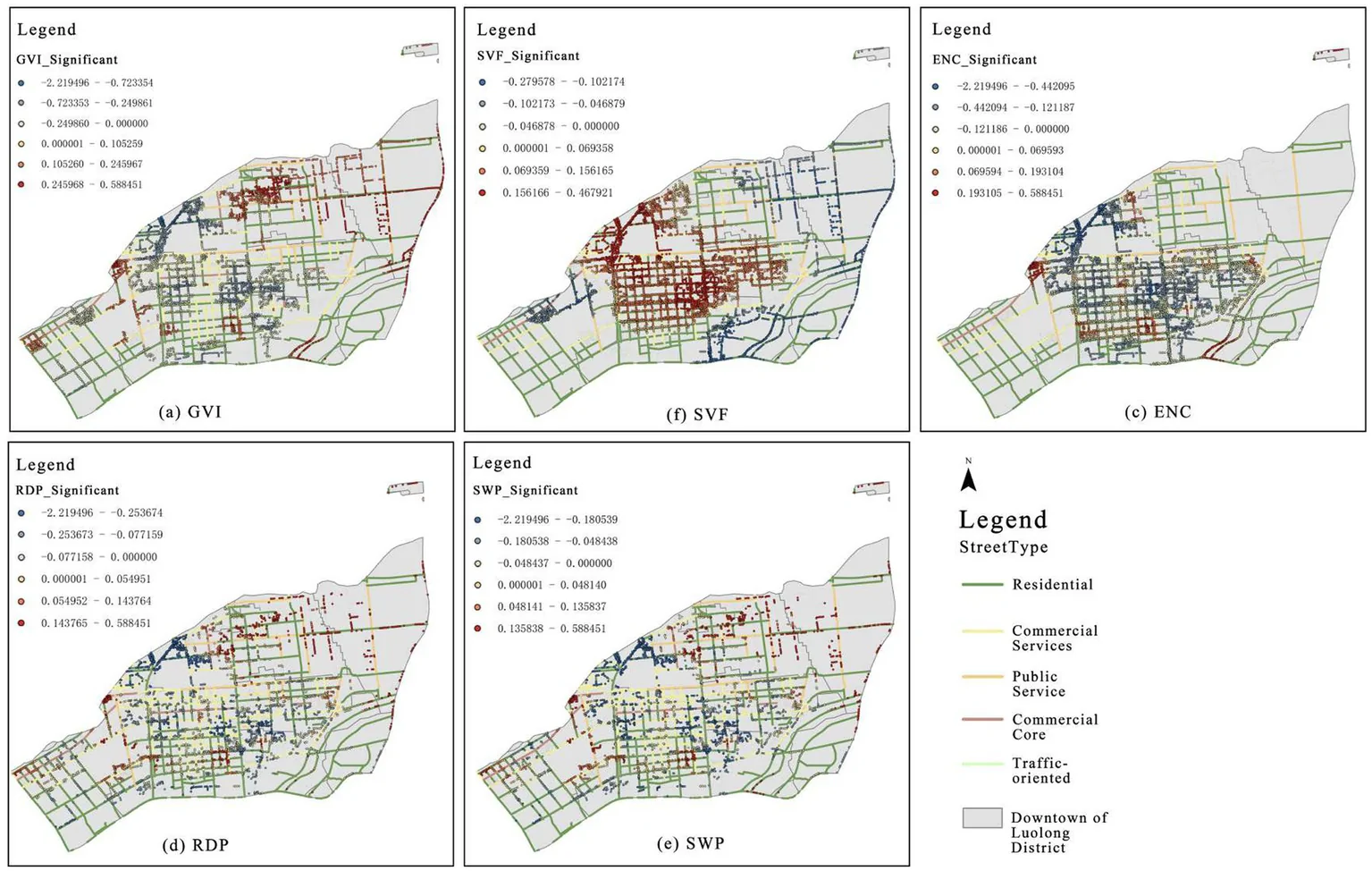
Spatial pattern diagram of local coefficient of street view indicators.

The statistical distributions of local coefficients in Figure 9 clearly demonstrate that the spatial dispersion of different independent variables is associated with their corresponding spatial bandwidths. RDP and SWP, which exhibit micro-scale bandwidths, display the widest and longest box ranges among all variables in the boxplots, confirming their pronounced spatial variability at the micro-scale. In contrast, SVF and ENC, which possess macro-scale bandwidths, show the narrowest and most compact box structures, indicating relatively stable spatial variation patterns.

In the spatial pattern maps of local coefficients for street-view metrics (Figure 10), only statistically significant local coefficients with |t| > 1.96 were retained to ensure coefficient robustness (30).

The effects of individual spatial metrics on LST exhibited significant spatial differences, as follows:

The local effects of RDP varied considerably, and RDP was positively associated with cooling in most commercial center-type and commercial service-type streets. The cooling benefits of GVI were mainly concentrated in commercial service-type and public service-type streets. SVF was positively associated with warming in commercial center-type, commercial service-type, and public service-type streets. In traffic-oriented streets, SVF showed a negative association with warming, but this relationship was not statistically significant. The local regulatory effect of ENC on temperature was most pronounced in commercial center-type and commercial service-type streets. Except in commercial service-type streets, SWP was positively associated with cooling across the other street types.

### 3.3 Thresholds and turning points of street-view metrics in relation to land-surface heat exposure

#### 3.3.1 Contribution ranking and heterogeneity of street-view metrics in explaining LST

To explain the contribution magnitude and direction of the effects of street-view metrics on summer land-surface heat exposure in Luolong District, XGBoost-SHAP was applied to all 9,157 sampling points in the study area for global feature analysis and additive feature attribution analysis (Figure 11). The bar chart on the left ranks the street-view metrics in descending order according to their global mean absolute SHAP values, while the nonlinear scatter plot on the right shows the distribution of individual sample points.

**Figure 11:**
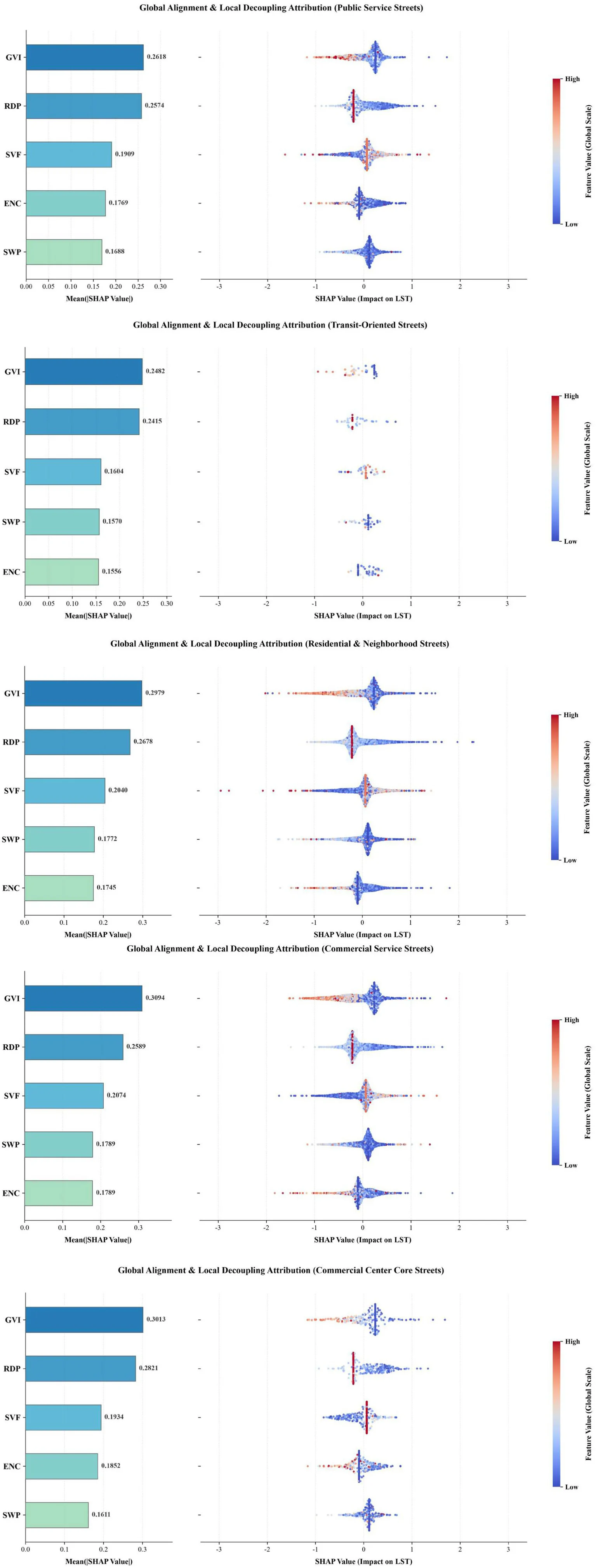
SHAP dual axis linkage composite diagram.

As shown in the bar chart, RDP ranked first in contribution strength across almost all street types, indicating that hardened road surfaces exerted the strongest positive driving effect on the increase in summer land surface temperature. GVI ranked second in contribution strength, highlighting the central role of three-dimensional street vegetation in microclimate regulation and the cooling process.

The scatter plot shows that SVF was generally positively associated with land-surface heat exposure on streets. The high feature values of RDP were all distributed to the left of the zero axis, indicating that large areas of motor vehicle lanes produced a cooling effect. The high and low feature values of GVI were distributed on both sides of the zero axis, suggesting that the cooling effect of high GVI on streets may have a potential threshold. ENC was generally negatively associated with land-surface heat exposure on streets.

#### 3.3.2 Thresholds and turning points of street-view metrics affecting LST

Based on the contribution magnitude and direction of each street-view metric, SHAP scatter plots were generated for each type of street to identify the cooling thresholds and maximum cooling benefit points for urban microclimate optimization. The scatter plots illustrate the nonlinear relationships between the street-view metrics and land-surface heat exposure, with the Y = 0 baseline serving as the boundary between warming and cooling, as shown in Figure 12. First, the LOWESS algorithm was used to extract the overall physical trends of the SHAP scatter points. To avoid interference from a small number of outlier samples, geometric optimization was performed within the 5–95% confidence interval of the data distribution. Second, the maximum cooling benefit point represents the point at which the maximum cooling benefit is achieved. To scientifically identify the maximum cooling benefit point, the algorithm aimed to find the optimal balance position for cooling efficiency. For most curves, the position where the cooling effect reached 85% of its maximum value was directly extracted. For more complex curves, the turning point where the cooling effect began to weaken substantially was extracted. This criterion ensures that streets can obtain most of the cooling benefits while avoiding excessive occupation of urban space in pursuit of maximum cooling.

**Figure 12:**
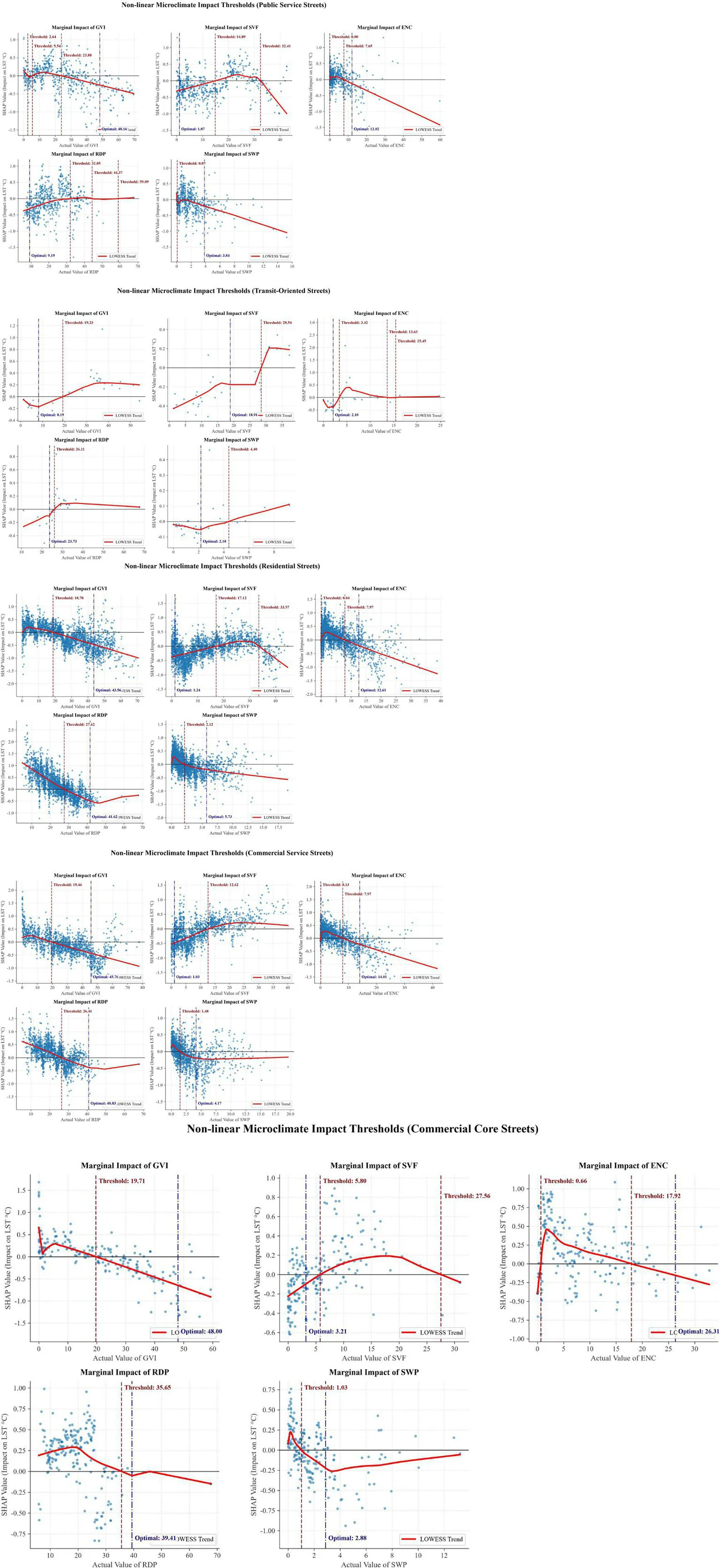
SHAP scatter plot and cooling threshold recognition.

It should be noted that the concepts of “cooling threshold” and “maximum cooling benefit point” defined in this section are essentially statistical thresholds of marginal contributions derived from the XGBoost-SHAP model. They reflect the mathematical turning points of the effects of street-view metrics in machine learning predictions, rather than strict thresholds of physical mechanisms underlying the microclimate. Their use as broad references for urban planning is intended to provide data-driven empirical guidance.

As can be seen from the figure, for the vast majority of streets, GVI was positively associated with land-surface heat exposure at relatively low values, and only after crossing specific thresholds (e.g., 20.41 for residential and daily-life service streets and 16.84 for commercial service-type streets) did it stably enter a negative association. The scatter plots of the other street-view metrics showed relatively clear positive–negative transition points. It is worth noting that RDP in commercial center-type streets exhibited a distinctive pattern of sustained warming due to the influence of the extremely high-density urban built environment.

## 4 Discussion

### 4.1 Significant spatial differences in the potential associations between street-view metrics and land-surface heat exposure on streets

It should be noted that the nonlinear thresholds extracted by the XGBoost-SHAP model in this study are essentially statistical inferences rather than physical causal relationships. The microclimate mechanisms, such as “convection” and “transpiration,” discussed in Table 7 are interpretations of the statistical patterns based on existing theories, rather than simulation results from a fluid dynamics model. Meanwhile, the discussion of the cooling thresholds of each street-view metric must take into account the local geographical characteristics and climatic conditions of Luoyang. Luoyang is located in a basin that is high in the northwest and low in the southeast, with a dustpan-like shape. Calm or light-wind conditions frequently occur in summer, resulting in relatively poor air circulation. These specific climatic conditions have a profound influence on the cooling effects of the individual metrics (Table 7):

**Table 7:** Threshold and turning point of thermal contribution of street view indicators.

Street type	Street-view metrics	Cooling threshold	Golden temperature control point	The physical mechanism of microclimate
Public Service	GVI	≥23.88%	48.16%	Plant leaf shading and transpiration cooling initiation
	SVF	≤14.89% or ≥32.41%	1.07%	Extremely low SVF effectively intercepts direct solar radiation during the daytime, playing a dominant role in shading and cooling
	ENC	≥7.65%	12.02%	Continuous shadows formed during the day
	RDP	≤32.05%	9.19%	Moderately reducing road width can reduce the physical heat storage of asphalt pavement
	SWP	≥0.07%	3.84%	Under large areas of hardened surfaces, the proportion of pedestrian pavement should be precisely controlled to reduce heat capacity
Transit-Oriented	GVI	≤19.23%	8.19%	Avoid overly dense tree canopies blocking light winds and prevent traffic exhaust and heat from accumulating under trees
	SVF	≤28.54%	18.91%	Maintain a relatively high degree of openness and use natural winds to disperse heat emissions from motor vehicles
	ENC	≤3.42%	2.10%	Maintain an open street interface to ensure unobstructed airflow and prevent sensible heat from traffic from accumulating due to obstruction by structures along the street
	RDP	≤26.11%	23.73%	An appropriate road proportion maintains an unobstructed convective ventilation corridor to remove anthropogenic heat
	SWP	≤4.40%	2.18%	The heat storage effect of pedestrian pavement is significant, and unobstructed hardened pedestrian paths should be minimized
Residential and Neighborhood	GVI	≥18.78%	43.56%	Shading from plant leaves and cooling through transpiration begin to take effect
	SVF	≤17.12% or ≥33.57%	1.24%	The deep street-canyon effect provides natural shading and prevents residential areas from being exposed to intense daytime solar radiation
	ENC	≥7.97%	12.61%	Continuous shadows formed during the day
	RDP	≥27.62%	41.62%	Wide roads form ventilation corridors that carry away heat from the road surface
	SWP	≥2.12%	5.73%	Lateral building shadows and continuous green shade coverage enable pedestrian spaces to achieve a cooling effect
Commercial Service	GVI	≥19.46%	45.76%	Large-scale tree canopy networks are key to reducing heat from hardened surfaces in commercial areas
	SVF	≤12.62%	1.03%	Extremely low SVF effectively intercepts direct solar radiation during the daytime, playing a dominant role in shading and cooling
	ENC	≥7.97%	14.01%	An appropriate height of commercial podium buildings provides effective shading for commercial activities at the ground level
	RDP	≥26.41%	40.83%	Wide roads form ventilation corridors that carry away heat from the road surface
	SWP	≥1.48%	4.17%	Affected by both artificial heat and high-rise obstruction, the golden point for cooling has shifted to the right
Commercial Center Core	GVI	≥19.71%	48.00%	Strong heat-island conditions need to be overcome through ecological cooling achieved by an extremely high GVI
	SVF	≤5.80% or ≥27.56%	3.21%	Extremely low SVF effectively intercepts direct solar radiation during the daytime, playing a dominant role in shading and cooling
	ENC	≥17.92%	26.31%	High-rise building facades provide sufficient shade and significantly reduce near-ground land-surface heat exposure
	RDP	≥35.65%	39.41	Asphalt pavement within the CBD is a persistent warming factor, and no conventional cooling turning point can be identified
	SWP	≥1.03%	2.88%	High-rise buildings severely obstruct wind, making pedestrian pavement highly susceptible to heat storage

First, calm weather limits the cooling effect of vegetation. Vegetation mainly absorbs surrounding heat through leaf transpiration. Under the low wind speeds that occur in Luoyang in summer, for public service, commercial service, and residential and daily-life service streets, GVI needs to reach a relatively high threshold (above 18.78% ~ 23.88%) before its cooling effect becomes apparent, and their maximum cooling benefit points are all as high as 43.56% ~ 48.16%. However, for traffic-oriented streets, if GVI is too high, it not only blocks the weak natural wind but also makes the air under the tree canopy humid and stuffy (31). Therefore, when GVI exceeds 19.23%, it instead intensifies warming, and the maximum cooling benefit point is identified at only 8.91.

Second, the basin topography makes nighttime heat dissipation difficult. The basin topography itself is not conducive to the outward dissipation of heat. In most residential and commercial areas, an extremely low SVF (with the maximum cooling benefit point ranging from 1.03 to 1.24) can effectively block the strong shortwave radiation during the daytime. However, in traffic-oriented streets, due to the presence of substantial anthropogenic heat sources from traffic, a higher SVF (with the maximum cooling benefit point of 18.91) is required to maintain spatial openness and avoid heat accumulation (32).

Third, overly dense buildings can block weak natural ventilation. During the low-wind summer, urban heat dissipation depends on very weak air movement. Once ENC on both sides of the street is too high, dense building groups can obstruct air movement. However, the requirements differ among different streets: because commercial center-type streets need to provide shading from high-intensity solar exposure, their maximum cooling benefit point for ENC reaches 14.01; whereas in traffic-oriented streets, ENC enters the warming range after crossing the low threshold of 3.42 (32).

Fourth, the positive ventilation effect of wide roads and the heat storage of hardened pavement. One of the most significant findings of this study is that RDP in commercial center-type streets remained entirely within the positive warming range before 35.65. This objectively confirms that, in the densely built core area of the CBD, asphalt pavement is highly positively associated with land-surface heat exposure, and only when the road is extremely wide and forms an obvious ventilation corridor does it have a slight potential for cooling (33). In other types of streets, RDP and SWP showed different benefits of advective heat dissipation after crossing specific thresholds (31).

### 4.2 Differentiated optimization strategies for multiple street types based on land-surface heat exposure risk classification

After analyzing the impact thresholds and maximum cooling benefit points of street-view metrics for different types of streets, a research framework of “risk diagnosis–priority assessment–strategy development” was established for each type of street in Luolong District to intuitively diagnose the microclimate problems of typical street areas in Luolong District and quantify the range of future optimization.

At the risk diagnosis stage, a matrix of the current status and sensitivity of street-view metrics was constructed (Figure 13). The left panel (a), the current status diagnosis, shows the actual average levels of six street-view metrics across five different street types in Luolong District, strictly defined according to the nonlinear “cooling thresholds” and “maximum cooling benefit points” extracted from the XGBoost-SHAP model in the previous analysis. It directly reveals the metrics that exceed the thresholds for each type of street and the extent to which they exceed them. The right panel (b), the sensitivity diagram, shows the sensitivity of the street-view metrics. In this study, the “mean” of the MGWR coefficients was used as the sensitivity evaluation criterion because the streets had already been classified by function using the K-means algorithm in the previous stage, and the anthropogenic heat emissions and locational characteristics of streets within the same type are highly similar. Therefore, using the average coefficients of streets within the same type as the representative measure of sensitivity can not only effectively reduce the interference of within-group differences, but also address the difficulty of developing individual strategies for each street in actual urban planning. This approach of “classification first, followed by averaging” takes into account both the accuracy of scientific analysis and the practicality of planning implementation.

**Figure 13:**
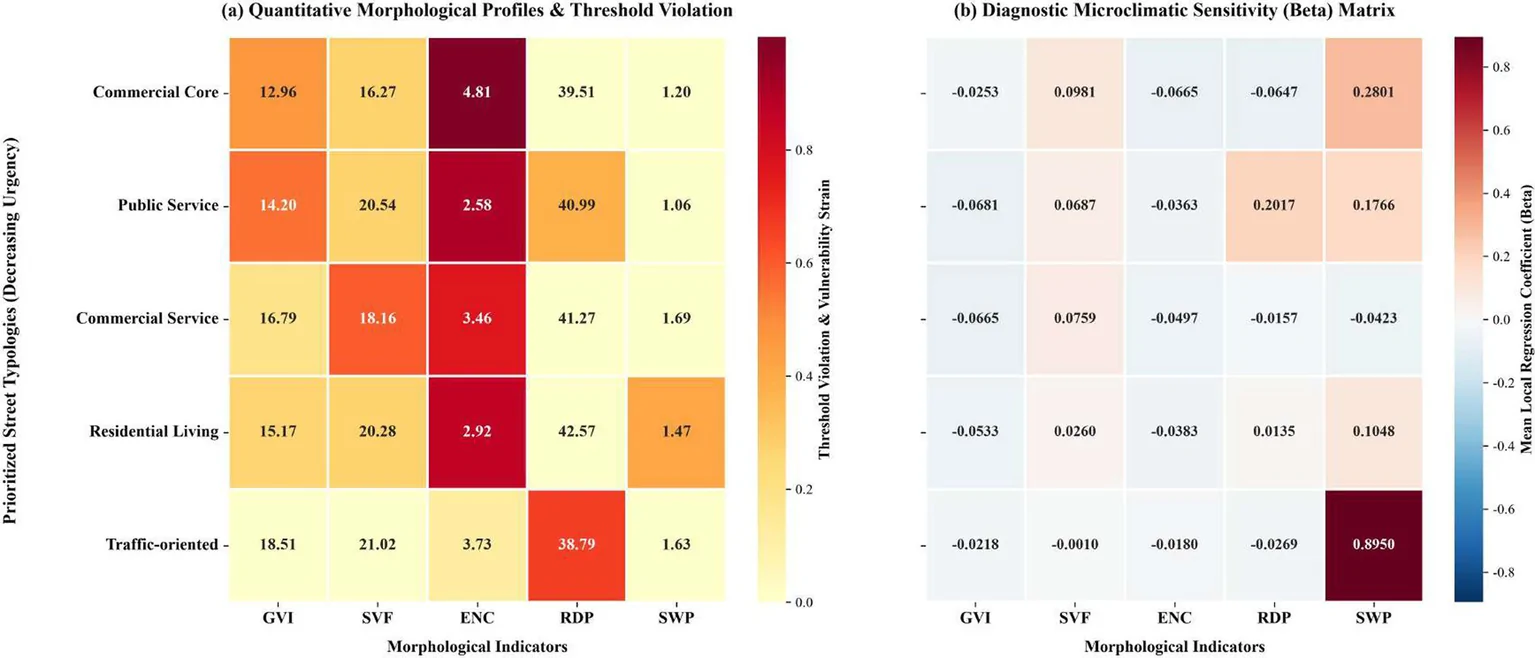
Current situation and sensitivity matrix of street view indicators. **(a)** Quantitative morphological profiles & threshold violation; **(b)** diagnostic microclimatic sensitivity (beta) matrix.

The criteria for determining the risk levels of land-surface heat exposure are mainly based on the following three standards. First, the number of street-view metrics exceeding the threshold within each street type is counted. If both the three-dimensional spatial form and the two-dimensional underlying surface exceed the thresholds, compound heat is formed, increasing the difficulty of mitigation and resulting in a higher risk level. Second, referring to the right panel (b), the more positive the β value of an over-threshold metric within a street type is, the more sharply the LST increases with each deterioration of that metric, resulting in a higher risk level. Third, from the perspective of the practical feasibility of planning implementation, a distinction is made between “hard indicators that must be controlled through macro-level regulatory planning” and “soft indicators that can achieve rapid results through micro-level improvement.”

Based on the above matrix and classification criteria, differentiated improvement strategies were proposed for the five street types (Table 8).

**Table 8:** Targeted optimization strategy based on street types.

Street type	Typical block name	Indicator characteristics	Realistic pictures	Problem Identification	Optimization Strategy
Public Service	Binhe South Road Service Road	Extremely low GVI (≤ 3%)	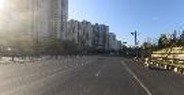	The measured LST in this type of street area approaches 39 °C. Its core problem is that GVI is close to 0%, while SVF shows a polarized pattern in Tunnel Road and Zhanlan Road. During the daytime, hardened surfaces are exposed to intense solar radiation, while the cooling and shading effects of vegetation transpiration are weak.	SVF is reduced to 1.07%, with a mean cooling effect of 0.277 °C and a maximum local cooling effect of 2.631 °C. GVI is precisely set at its maximum cooling benefit point of 48.16%, with a mean cooling effect of −0.002 °C and a maximum local cooling effect of 0.098 °C.
	Changxia Men Tunnel		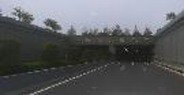		
	Zhanlan Road		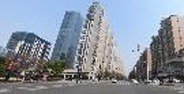		
Transit-Oriented	Tongqu Road	Low GVI (≤ 5%) SVF is around 25%	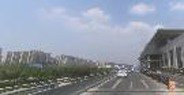	These streets have a relatively large area of hardened road surfaces. Particular attention should therefore be paid to GVI and SVF to alleviate the problem of heat accumulation on asphalt surfaces during the daytime and its difficulty in dissipating at night.	GVI is strictly controlled at around 19.23% to prevent heat accumulation. SVF is maintained at its maximum cooling benefit point of 18.91% to ensure unobstructed ventilation at the macro scale.
			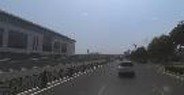		
			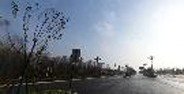		
Residential and Neighborhood	Longmen Avenue	High SVF (>30%) High lane ratio (RDP = 67.67%)	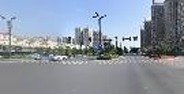	The high heat storage caused by the wide hardened traffic surfaces reduces the thermal comfort of nearby residents during walking, while effective continuous tree-canopy shading is lacking.	SWP is reduced to its maximum cooling benefit point of 5.73%, with a mean cooling effect of −0.044 °C and a maximum local cooling effect of 0.965 °C. GVI is increased to the optimal ecological range of 43.56%, with a mean cooling effect of −0.002 °C and a maximum local cooling effect of 0.184 °C.
	Yiluo East Road		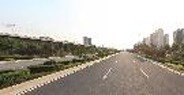		
	Zhoushan Avenue		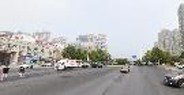		
Commercial Service	Cuiyun East Road	High lane ratio (RDP = 67.67%) Low GVI (GVI ≤ 10.52%)	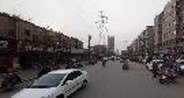	The interaction between commercial pedestrian flows and large areas of exposed heat-absorbing asphalt surfaces, together with insufficient GVI, leads to deterioration of the microclimate.	SWP is reduced to its maximum cooling benefit point of 4.17%, with a mean cooling effect of −0.055 °C and a maximum local cooling effect of 1.583 °C. GVI is increased to the optimal ecological range of 45.76%, with a mean cooling effect of −0.001 °C and a maximum local cooling effect of 0.375 °C.
	Taikang Road		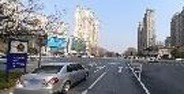		
	Binhe South Road		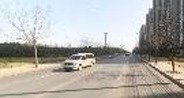		
Commercial Center Core	Zhenghe Road	Extremely high green visual acuity (GVI ≈ 22.68–59.28%)	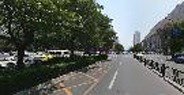	Motor vehicle lanes within the CBD are uncontrollable pure heat sources. High-density tree canopies and high-intensity buildings simultaneously obstruct natural ventilation within the street canyon.	ENC is increased to 26.31%, with a mean cooling effect of 0.133 °C and a maximum local cooling effect of 0.952 °C. GVI is increased and controlled at around 48.0%, with a mean cooling effect of 0.033 °C and a maximum local cooling effect of 1.435 °C.
	Luoyi Road		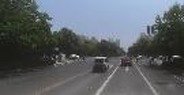		
	Garden Street		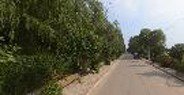		

#### 4.2.1 Commercial center core streets — highest priority level

As shown in Figure 13A, Commercial Center Core Streets exhibit multiple overlapping above-threshold conditions. Their GVI is substantially below the 19.71% cooling threshold, and ENC is severely insufficient. Figure 13B further reveals their microclimatic vulnerability: SVF show the highest sensitivity values among all street types, indicating that even minor increases in radiation exposure can trigger substantial warming effects in these streets.

Given that the urban fabric and highly impervious underlying surfaces of commercial center streets are already highly established, large-scale demolition and reconstruction should be avoided. Instead, optimization strategies should focus on adaptive retrofitting (Figure 14A):

(1) Using the sensitivity characteristics of SVF and ENC, continuous cantilevered arcades and tensile membrane shading canopy networks are introduced along the ground-floor commercial frontage, increasing ENC to the cooling maximum cooling benefit point of 26.31%, with a maximum local cooling effect of 0.952 °C, thereby alleviating the warming caused by insufficient ENC (34).
(2) “Three-dimensional suspended green walls” and “cantilevered rooftop greening” are implemented on building facades (35). Given the strong wind-blocking effect of buildings along these streets, more greening is not necessarily better. The strategy should use 19.71% as the baseline cooling threshold and control GVI below the ecological saturation point of 48%. The maximum local cooling effect can reach 1.435 °C, thereby avoiding further disruption of natural ventilation within the street canyon caused by overly dense tree canopies.
(3) Misting cooling sources, highly dense green islands, and wetland landscape water features are arranged at equal intervals in areas with high pedestrian concentrations (36). Through active humidification and localized heat exchange, cooling within the street canyon can be achieved without altering the overall building framework.

**Figure 14:**
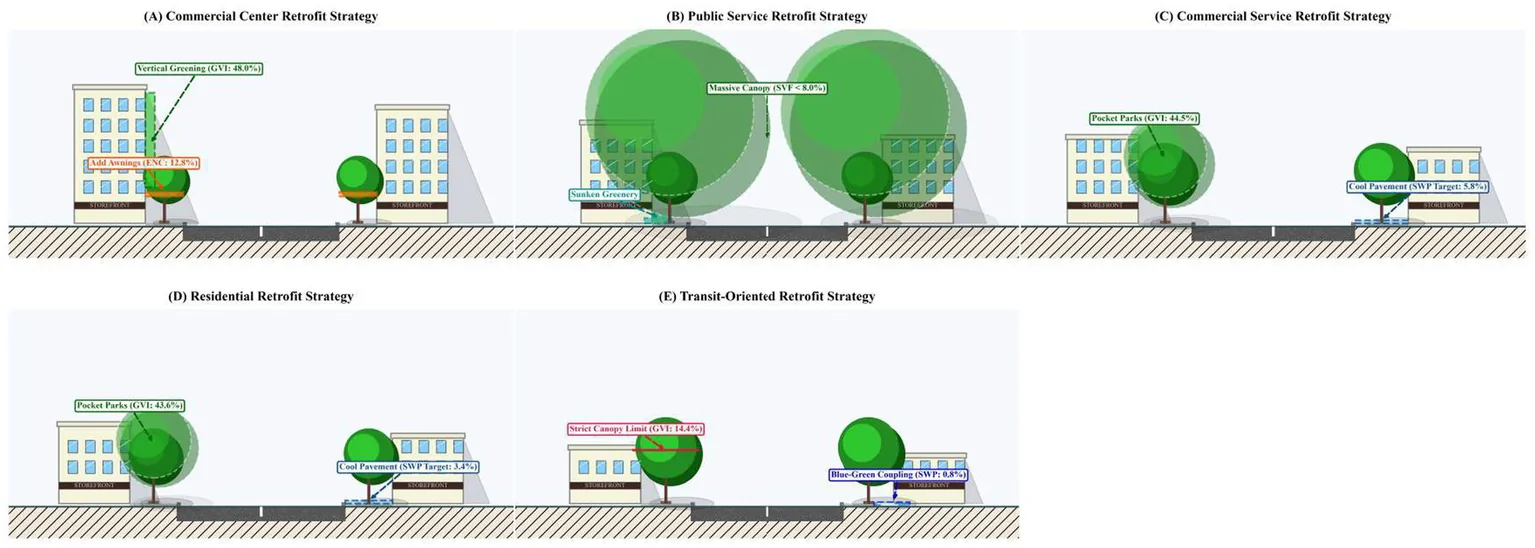
Sectional map of various types of street renovation. **(A)** Commercial center retrofit strategy; **(B)** Public service retrofit strategy; **(C)** Commercial service retrofit strategy; **(D)** Residential retrofit strategy; **(E)** Transit-oriented retrofit strategy.

#### 4.2.2 Public service streets — second-highest priority level

Public service-type streets mainly connect administrative, research, and medical institutions and are areas of frequent activity for sensitive groups such as older adults and children. Figure 13A shows that ENC is the lowest across the entire study area, while the warming sensitivity of RDP is extremely high, indicating that wide, hardened surfaces with insufficient shading are the main cause of heat accumulation in summer. Therefore, planning should adopt the strategy of “removing walls and introducing greenery, and reducing hardened surfaces within large institutional compounds” (Figure 14B):

(1) Optimizing hardscape and surface materials: Reduce unnecessary hardened areas in plazas and parking lots and convert them into continuous sunken green spaces, grassed swales, or rain gardens. For the remaining paved areas, promote the use of high-albedo cool pavement materials and permeable ecological bricks to alleviate the land-surface warming effect caused by excessive RDP (37).
(2) Enhancing the continuity of shading in pedestrian spaces: In response to the current high SVF, deciduous trees with full canopies and dense foliage should be planted along building setback spaces and sidewalks to ensure that pedestrian spaces can receive continuous tree-canopy coverage at noon in summer (38). Scenario simulations show that when local SVF is reduced to below 8% through continuous tree-canopy coverage, the maximum local land-surface heat exposure reduction can reach 2.631 °C, with an improvement of 0.277 °C in the regional average. Combined with increasing GVI to its maximum cooling benefit point of 48.16%, a maximum local cooling effect of 0.098 °C can be achieved.

#### 4.2.3 Commercial service streets and residential & neighborhood streets — moderate priority level

These two types of streets are conventional street areas and are classified as moderately important. Their overall spatial forms are relatively balanced, with all indicators falling within reasonable ranges. Their GVI values are 19.46 and 18.78, respectively, which are also very close to the cooling threshold line. Meanwhile, the sensitivity characteristics of the various street-view metrics are relatively moderate, and the spatial structure is relatively stable. Therefore, these types of streets do not require large-scale spatial restructuring; the cooling objective can be achieved through only local greening improvements and minor adjustments to the underlying surface (Figures 14C,D):

(1) Building a continuous green ecological network: Given that the cooling effect of GVI has spatial continuity, scattered point-based greening should be avoided. Pocket parks along the streets should be used for greening improvements, not only to quickly cross the cooling threshold but also to move toward the maximum cooling benefit points of 45.76 and 43.56%. When GVI is increased to the maximum cooling benefit points, the maximum local cooling effects in residential and commercial areas reach 0.184 °C and 0.375 °C, respectively, forming continuous community cool islands (39).
(2) Optimizing the microclimatic environment of pedestrian spaces: Given the sensitivity of SWP to the thermal environment, local improvements can be implemented in existing hardened pedestrian paths by “replacing them with cool pavement and increasing tree-trunk shading.” SWP in residential and commercial areas should be precisely controlled near the maximum cooling benefit points of 4.17 and 5.73%, respectively. The maximum cooling effects in local high-heat areas can reach 0.965 °C in residential areas and 1.583 °C in commercial areas, respectively, thereby cutting off the deterioration of the microclimate at its source (40).

#### 4.2.4 Transit-oriented streets — low-intensity improvement level

As major traffic corridors carrying the primary transportation function, these streets have a relatively good basis for heat dissipation in terms of their overall spatial form. Figure 13 shows that the relatively large road proportion forms a good ventilation corridor, which can effectively dissipate the heat accumulated on road surfaces through air convection. However, these streets have relatively high SVF, leaving the overall space exposed to solar radiation. Particularly in the absence of tree shading, SWP has an extremely high warming sensitivity, and the exposed hardened surfaces on both sides of the street are highly susceptible to heat accumulation, becoming concentrated areas of land-surface heat exposure (41).

Overall, because these streets have good conditions for ventilation and heat dissipation, there is no need for large-scale adjustments to their roadway form, and their land-surface heat exposure risk level is the lowest among all street types. Therefore, future optimization strategies should focus primarily on improving greening in the pedestrian spaces on both sides of the road (Figure 14E):

(1) Preventing the accumulation of sensible heat from traffic: Given the specific characteristics of these streets, dense street trees should not be added blindly. GVI should be strictly controlled at around the maximum cooling benefit point of 8.19% to prevent dense tree canopies from obstructing the dissipation of vehicle exhaust and sensible heat from traffic (42).
(2) Optimizing the ventilation corridor and reducing wind resistance: Maintain a high degree of openness with SVF at around 28.54%, and strictly control ENC at 2.10% to avoid continuous solid walls, thereby reducing the surface roughness of the street and improving ventilation and heat-exchange efficiency (43).
(3) Strengthening the coupling of “blue-green” ecological cooling sources: For traffic corridors adjacent to wetland water bodies, continuous shelterbelts can be used to direct water evaporation toward pedestrian areas, while reducing exposed hardened pedestrian paths and controlling SWP at its maximum cooling benefit point of 2.18% (44).

### 4.3 Multilevel impact perspectives on street land-surface heat exposure research

This study overcomes the limitations of traditional urban thermal environment research, which often overlooks spatial differences and nonlinear mechanisms. By using K-means clustering to classify street types, the study adopts a “global training and category-specific extraction” approach, effectively avoiding model distortion and overfitting caused by small sample sizes. On this basis, the land-surface heat exposure risk levels of different street types were identified, and targeted, refined optimization strategies for urban streets were proposed.

However, the formation and evolution of street microclimates are by no means determined solely by a single physical parameter or a fixed underlying surface, but rather result from the physical synergy of 2D and 3D morphological factors and the multidimensional effects of land-surface heat exposure and human subjective thermal perception. It should be noted that this study currently focuses mainly on cooling mechanisms at the objective physical level. However, a complete research framework for street land-surface heat exposure should extend beyond physiological boundaries to the level of psychological perception in the future. For example, psychological heat tolerance, perceived coolness, and the sense of safety. This section further analyzes the combined potential relationships from multiple dimensions, including physical space, social behavior, and psychological perception, with the aim of providing a broader theoretical perspective for interdisciplinary research on urban microclimates and healthy living environments (45).

#### 4.3.1 Synergistic effects of 2D underlying surfaces and 3D street canyon morphological factors

To reveal the synergistic effects of 2D underlying surfaces and 3D spatial morphology on urban LST, SHAP interaction values were employed to quantify the nonlinear interaction effects between the 2D indicators (RDP, and SWP) and 3D indicators (SVF, GVI, and ENC) across the study area. The three-dimensional response surfaces of the interaction matrix are presented in Figure 15.

**Figure 15:**
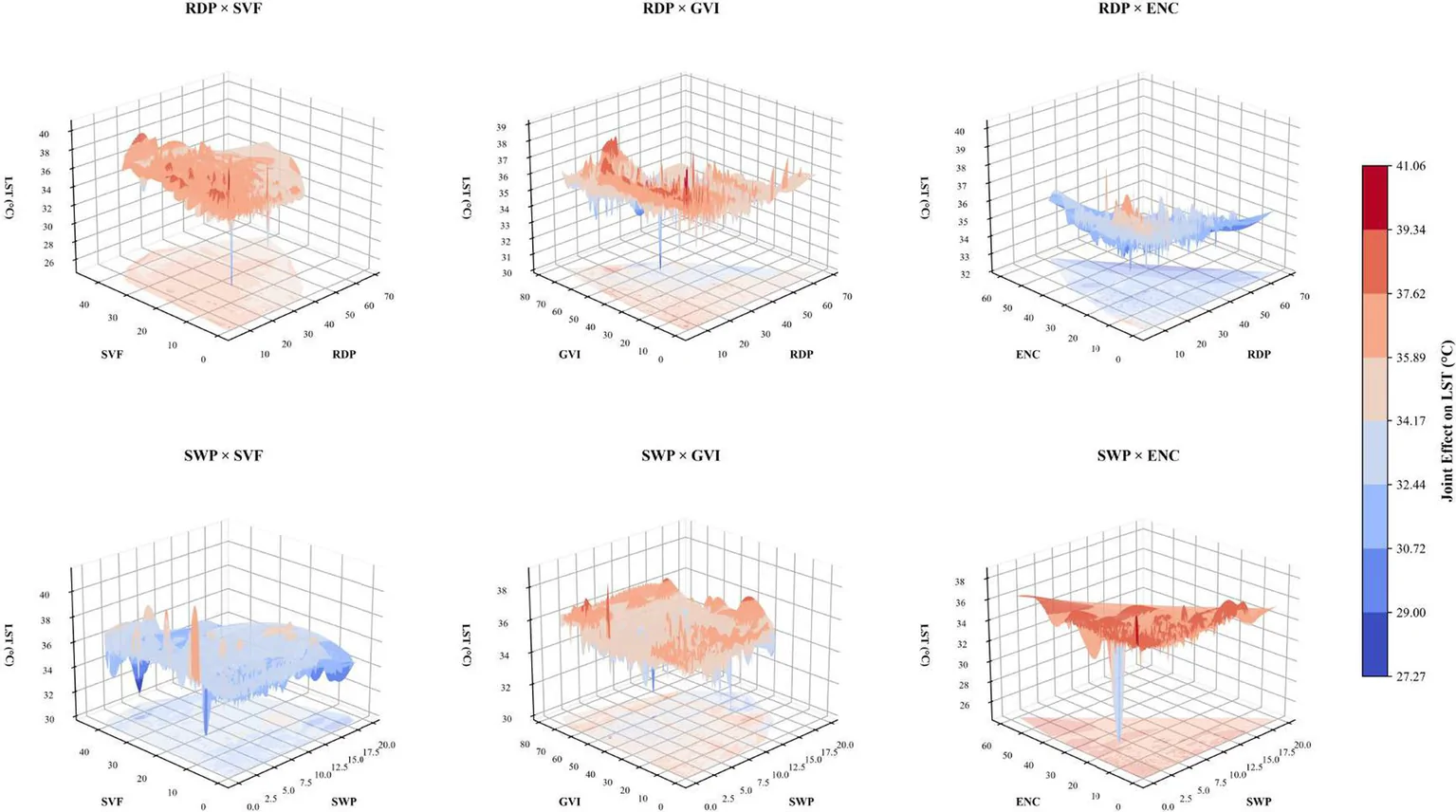
The interactive impact of 2D and 3D metrics on LST.

As shown in the matrix, the two groups of metrics, RDP × ENC, and SWP × SVF, show cooling benefits under most interaction conditions. The interaction between RDP × GVI shows a balanced effect on the thermal environment. It is recommended that GVI be maintained within the corresponding cooling threshold for each street type at low RDP levels, so as to achieve a dynamic balance between using shade for cooling and maintaining unobstructed ventilation corridors.

In contrast, the three interaction combinations of RDP × SVF, SWP × GVI, and SWP × ENC generally demonstrate warming effects under most interaction conditions, indicating that microclimate optimization requires coordinated control with other street-view indicators.

#### 4.3.2 Synergistic relationship between street land-surface heat exposure and citizens’ physical and mental health

The high-temperature environment resulting from high-density urban development not only challenges citizens’ physiological functions but also induces psychological stress and anxiety. Therefore, investigating the dual regulatory benefits of three-dimensional street-view indicators at both physiological and psychological levels is essential for designing resilient urban microclimates (46).

Abundant vegetation within streetscapes can not only achieve physical cooling through evapotranspiration but also provide cognitive buffering effects at the visual and psychological levels (47). This “visual coolness” generated by high GVI can effectively regulate negative emotions and significantly enhance citizens’ psychological thermal tolerance and acceptance of high-temperature environments. The building shadows generated by moderate ENC can reduce pedestrians’ exposure to thermal radiation while simultaneously providing a sense of security and shelter at the psychological level. Such moderate spatial enclosure effectively alleviates feelings of helplessness and emotional discomfort caused by exposure to intense sunlight in highly open environments.

Therefore, future planning practices should not be limited to achieving physical cooling objectives alone. Instead, they should maximize the psychological regulatory functions of street environments by creating continuous green landscapes and comfortable shaded spaces, thereby comprehensively enhancing the resilience of human settlements in high-density urban neighborhoods.

### 4.4 Reflection on research limitations

From the perspective of humanistic geography and climate-resilient urban planning, a single temperature indicator cannot fully represent the actual thermal perception of human skin in complex outdoor environments (48). Therefore, future research should move beyond purely physical temperature observations toward comprehensive biometeorological indicators such as PET and UTCI (49). This study used data collected only once during the summer, which can only demonstrate the correlation between street-view characteristics and temperature levels. Due to the lack of long-term tracking across seasons, a strict causal relationship cannot yet be established. Future research could incorporate long-term dynamic tracking data to further verify the actual cooling effects of street improvements. In addition, the models used in this study reflect statistical correlations rather than causal relationships. Future research could combine physical fluid simulation models, such as ENVI-met or CFD, for validation and further establish the physical mechanisms underlying street cooling.

## 5 Conclusion

Based on street-view images and multi-source spatial data, this study quantitatively confirms that LST at the block scale is not the product of a globally consistent microclimate process, but is highly constrained by the spatial heterogeneity and nonlinear turning-point effects of street landscape indicators.

From the perspective of algorithms and spatial methodology, this study combined the synergistic framework of “K-means clustering, MGWR spatial regression, and the XGBoost-SHAP decision tree model” to successfully explain the heterogeneity of potential influence relationships across spatial scales and identify the critical saturation points of environmental variables. From the perspective of scientific quantitative conclusions, this study precisely established the cooling ranges and maximum cooling benefit points. From the perspective of practical urban planning implementation, based on the above scientific findings, a set of highly spatially adaptive improvement strategies was developed for the five identified street types. The research approach established in this study not only clarifies the nonlinear effects of street landscapes on microclimates from a theoretical perspective, but also provides practical data support and design guidelines for mitigating local land-surface heat exposure and promoting refined street renewal in high-density urban areas.

## Data Availability

The original contributions presented in the study are included in the article/supplementary material, further inquiries can be directed to the corresponding author.
